# Radiopharmaceutical tracers for cardiac imaging

**DOI:** 10.1007/s12350-017-1131-5

**Published:** 2017-12-01

**Authors:** Osamu Manabe, Tatsuya Kikuchi, Arthur J. H. A. Scholte, Mohammed El Mahdiui, Ryuichi Nishii, Ming-Rong Zhang, Eriko Suzuki, Keiichiro Yoshinaga

**Affiliations:** 1 Department of Nuclear Medicine, Hokkaido University Graduate School of Medicine, Sapporo, Japan; 2 Department of Radiopharmaceutical Development, National Institutes for Quantum and Radiological Science and Technology, National Institute of Radiological Sciences, Chiba, Japan; 3 Department of Cardiology, Leiden University Medical Center, Leiden, The Netherlands; 4 Diagnostic and Therapeutic Nuclear Medicine, National Institutes for Quantum and Radiological Science and Technology, National Institute of Radiological Sciences, 4-9-1 Anagawa, Inage-Ku, Chiba, 263-8555 Japan

**Keywords:** Cardiovascular disease, positron emission tomography, radiopharmaceutical, single-photon emission computed tomography

## Abstract

Cardiovascular disease (CVD) is the leading cause of death and disease burden worldwide. Nuclear myocardial perfusion imaging with either single-photon emission computed tomography or positron emission tomography has been used extensively to perform diagnosis, monitor therapies, and predict cardiovascular events. Several radiopharmaceutical tracers have recently been developed to evaluate CVD by targeting myocardial perfusion, metabolism, innervation, and inflammation. This article reviews old and newer used in nuclear cardiac imaging.

**Electronic supplementary material:**

The online version of this article (10.1007/s12350-017-1131-5) contains supplementary material, which is available to authorized users.

## Introduction

Cardiovascular disease (CVD) is the leading cause of death and disease burden around the world.^1^ Advances in single-photon emission computed tomography (SPECT) and positron emission tomography (PET), which allow for non-invasive imaging, are vastly improving the evaluation of myocardial perfusion and function.^2^,^3^ Nuclear cardiac imaging is useful to perform diagnosis and risk assessment and to monitor the impact of therapies through serial imaging. Several radiopharmaceutical tracers are used in nuclear cardiology imaging to target perfusion, metabolism, innervation, and inflammatory conditions. Nuclear imaging tests are suitable for almost all patients given the low possibilities of side effects from radiopharmaceutical tracers other than minimal radiation exposure. In this article, we will review SPECT and PET tracers used in assessing CVD.

## Tracers Used for Cardiac Imaging (Table 1)

### Inorganic Tracers

Inorganic compounds ^13^N-ammonia (^13^N-NH_3_) and ^15^O-water (^15^O-H_2_O) have been used for cardiac perfusion imaging.^4^ Both tracers are labeled with short-lived positron emitters (^13^N: 10 minute; ^15^O: 2 minute), which are therefore produced with an onsite cyclotron. ^15^O-H_2_O is freely diffused into cardiomyocytes. In contrast, the uptake mechanism of ^13^N-NH_3_ is unclear.^5^ Almost all ammonia molecules in the blood would be protonated to form NH_4_^+^ because of its pKa (9.3 at 25 °C). The ammonium cation would barely penetrate cell membranes to enter cardiomyocytes.

**Table 1:** Classification of cardiac imaging tracers by characteristics

Characteristics	Tracer
Inorganic tracers	^13^N-NH^3^
	^15^O-H_2_O
Radiometal ions	^201^Tl^+^
	^82^Rb^+^
	^67^Ga^3+^
	^18^F^−^
Small organic tracers	^11^C-acetic acid
	^11^C-palmitic acid
	^123^I-IPPA
	^18^F-FDG
	^123^I-BMIPP
	^18^F-FTHA
	^11^C-epinephrine
	^18^F-fluorodopamine
	Derivatives of guanethidine, metaraminol, and vesamicol
	Neuroreceptor ligands such as prazosin (α-blocker), carazolol (β-blocker) derivative, β-agonists (CGP12177 and CGP12388), and quinuclidinyl benzilate (anticholinergic compound)
	^11^C-PK11195 ^18^F-FEDAC
Radiometal complex tracers	^99m^Tc-sestamibi
	^99m^Tc-tetrofosmin
	Somatostatin analogs and annexin V tagged with ^64^Cu, ^68^Ga, or ^99m^Tc
	^99m^Tc-tagged annexin A5
	^111^In-oxine
	^99m^Tc-HMPAO

### Radiometal Ions

In addition to these inorganic compounds, several radiometal ions have been used as cardiac imaging tracers, especially in myocardial perfusion imaging. Initially, the monovalent cation of potassium-43 (^43^K^+^), a γ-emitter, was used for imaging of myocardial perfusion.^6^ However, the main gamma energy of this radionuclide (0.37 and 0.67 MeV) is somewhat too high for SPECT imaging. Also ^43^K has a relatively long half-life (22 hours) and emits relatively high-energy β-particles [300 keV (mean)]. K^+^ is actively transported into the myocyte by the cell membrane via Na^+^/K^+^ pumps. Therefore, other monovalent cations that emit γ-rays suitable for SPECT imaging were sought. The ionic radius of the candidate should be comparable to that of K^+^ (138 pm) to be a substrate of a Na^+^/K^+^ pump. The monovalent cation of thallium-201 (^201^Tl^+^, ionic radius; 150 pm) fulfills these requirements and has been widely used for diagnosis of coronary artery disease (CAD). Although ^201^Tl emits γ-rays of 135 and 167 keV, abundantly emitted characteristic x-rays (69 to 80 keV) are used for imaging.

A positron emitter, rubidium-82 (^82^Rb), has an ionic radius (152 pm) comparable to that of K^+^ in its monovalent cationic form (^82^Rb^+^) and belongs to the same family as K (alkaline metals). The kinetics of Rb^+^ are similar to those of K^+^ 7, and therefore, ^82^Rb^+^ has been widely used as a perfusion imaging tracer with PET in the United States (USA).^8^ In addition, the use of a positron-emitting isotope of K, potassium-38, has been also reported.^9^ Trivalent cations of gallium-67 (^67^Ga^3+^), a γ-emitter, have been used to detect inflammatory lesions. Ga^3+^ binds to ferric iron (Fe^3+^)-binding proteins such as transferrin and lactoferrin which are accumulated in inflammatory lesions.^10^ Besides cationic radionuclides, a monovalent anion of fluorine-18 (^18^F^−^) that is used for bone imaging has been used for imaging calcification lesions with PET.^11^

### Small Organic Tracers

Tracers of radiolabeled small organic compounds are used for imaging metabolism, synaptic function, and inflammation. In metabolic imaging, radiolabeled biomolecules and their derivatives are used. Biomolecules, acetic acid, and palmitic acid, substrates of oxygen metabolism and fatty acid metabolism, have been labeled with carbon-11 (^11^C-acetic acid and ^11^C-palmitic acid) and used for the assessment of respective myocardial metabolism.^12^ Iodophenylpentadecanoic acid labeled with iodine-123, (^123^I-IPPA) is also a substrate of fatty acid metabolism. For labeling with ^123^I, a phenyl group was incorporated into the structure of palmitic acid. In the development of tracers, derivatization of a biomolecule is often performed to obtain a compound that is metabolized by a certain metabolic step without undergoing further metabolism. 2-[^18^F]fluorodeoxyglucose (^18^F-FDG) is one such derivative of glucose. β-methyl-*p*-[^123^I]iodophenylpentadecanoic acid (^123^I-BMIPP) and [^18^F]fluoro-6-thia-heptadecanoic acid (^18^F-FTHA) introduce a methyl group and thioether in the alkyl chain, respectively, to terminate β-oxidation in the course of fatty acid metabolism.

In presynaptic cardiac imaging, a radiolabeled catecholamine and its derivative are also used as a tracer. ^11^C-labeled epinephrine and ^18^F-labeled fluorodopamine (^18^F-fluorodopamine) have been used to image the presynaptic sympathetic nervous system.^13^ In addition to biomolecules, xenobiotics including therapeutics are radiolabeled and used as tracers. Derivatives of guanethidine, metaraminol, and vesamicol are used for presynaptic imaging, and neuroreceptor ligands such as prazosin (α-blocker), carazolol (β-blocker) derivative, β-agonists CGP12177 and CGP12388, and quinuclidinyl benzilate (anticholinergic compound) derivatives are used for neuroreceptor imaging (Table 4).^13^

Radiolabeled receptor ligands for translocator protein 18 kDa (TSPO), peripheral-type benzodiazepine receptors, have also been used to image inflammation. TSPO is highly expressed in activated cells of the mononuclear phagocyte.^14^

### Radiometal Complex Tracers

Some tracers used in nuclear cardiology are radiometal complexes containing copper-64 (^64^Cu), gallium-68 (^68^Ga), or technetium-99m (^99m^Tc). They are classified into two groups. One contains those complexes that are used as tracers on their own. ^99m^Tc is used to form a complex with six methoxyisobutylisonitrile (^99m^Tc-sestamibi) and two 1,2-bis(di(2-ethoxyethyl)phosphino) ethane (^99m^Tc-tetrofosmin), which have been used for myocardial perfusion imaging. Their bulky structures contribute to reducing protein binding in the blood through steric hindrance. These tracers are positively charged (monovalent) but lipophilic. Therefore, they can be diffused into myocytes.

The other group includes complexes used as tags for peptides and proteins. Somatostatin analogs and annexin V tagged with ^64^Cu, ^68^Ga, or ^99m^Tc have been used for imaging symptomatic carotid atherosclerosis.^15^ ^64^Cu or ^68^Ga-tagged somatostatin analogs bind to somatostatin receptor subtype-2, which is upregulated in macrophages. ^99m^Tc-tagged annexin A5 binds to phosphatidylserine, which is externalized in apoptotic cells.

White blood cells enclosing radiometals, which are used for imaging infectious lesions, are prepared using lipophilic radiometal complexes. Indium-111 (^111^In) complexed with 8-hydroxyquinolines (^111^In-oxine) and ^99m^Tc complexed with exametazime (^99m^Tc-HMPAO) are diffused into the leucocyte. The subsequent dissociation of ligands results in enclosure of these radiometals in the cell.

## Radiotracers Categorized by Use

### Perfusion Imaging

**Figure 1:**
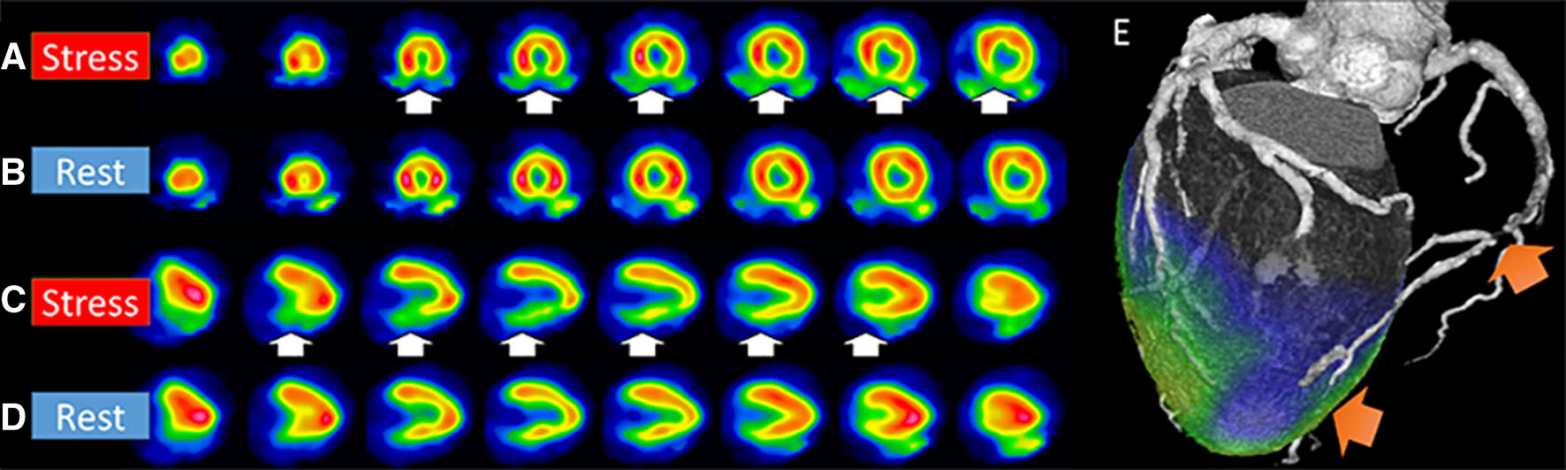
Myocardial perfusion images Perfusion images of short-axis image at stress (**A**) and rest (**B**), vertical long-axis image at stress (**C**) and rest (**D**) using ^99m^Tc-product, and fused image of stress perfusion and CT coronary angiography (CTCA; **E**) are displayed. Severe perfusion reduction is detected in the inferior wall at stress (white arrows). Fill-in is seen at rest indicating stress-induced ischemia in the right coronary artery (RCA). CTCA revealed significant stenosis in the RCA (orange arrows)

**Figure 2:**
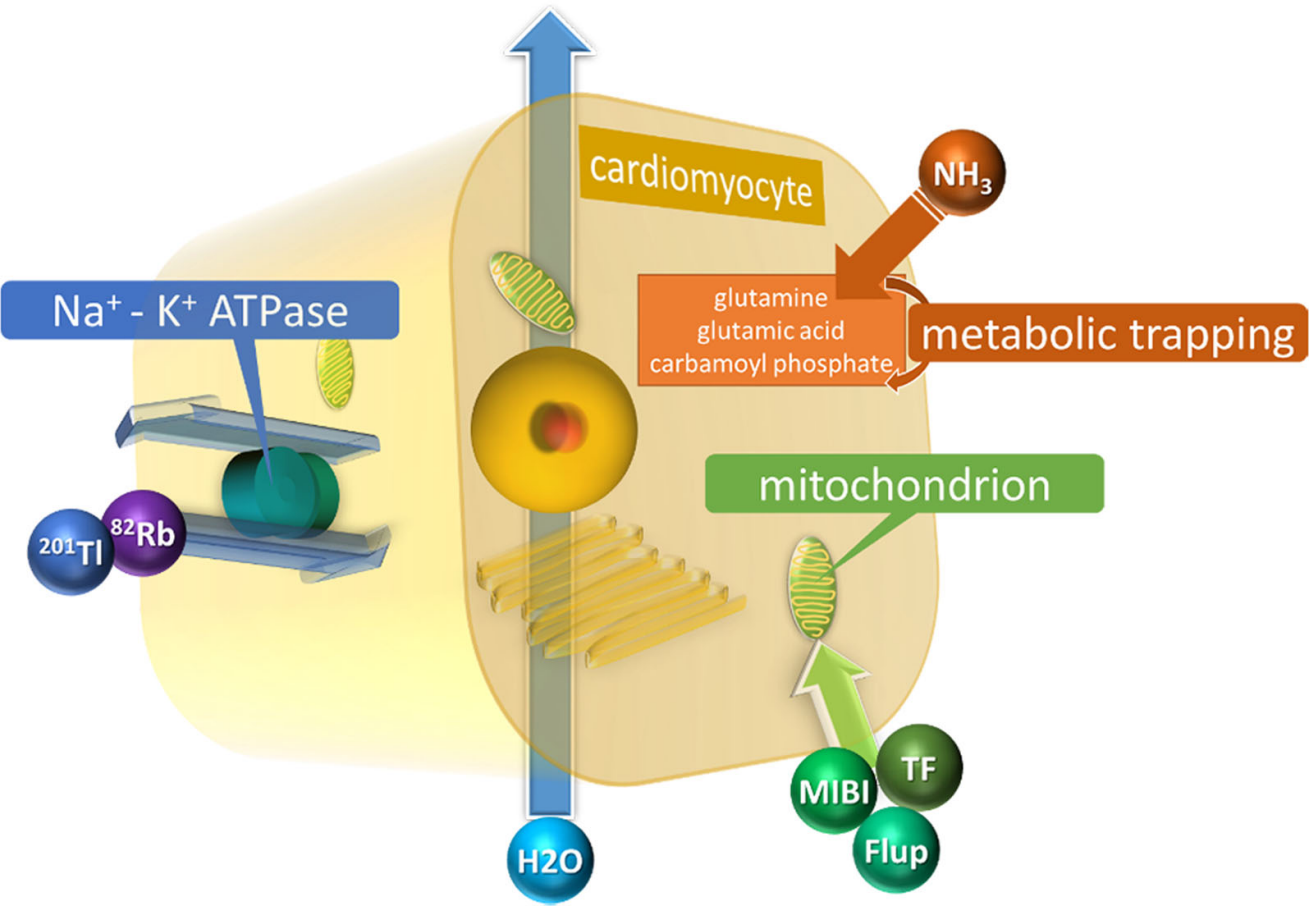
Schematic representation of tracers for assessing myocardial perfusion ^201^Tl and ^82^Rb are potassium analogs and are transported into the myocyte by cell membrane Na^+^/K^+^ pumps. Injected uptake of ^99m^Tc-sestamibi, ^99m^Tc-tetrofosmin, and ^18^F-flurpiridaz in the myocardium is related to the presence of intact mitochondria. The uptake mechanism of ^13^N-NH_3_ is unclear. After being taken into the myocyte, ^13^N-NH_3_ underwent metabolic trapping with the conversion of NH_3_ to glutamine, glutamic acid, and carbamoyl phosphate. ^15^O-H_2_O is metabolically inert and freely diffusible tracer

Myocardial blood flow (MBF) is supplied by coronary arteries to preserve adequate myocardial oxygen supply. At rest, coronary artery stenosis must exceed 85% to 90% of luminal diameter before there is a significant decrease of MBF. In contrast, maximal coronary flow has been shown to be reduced with stenosis of 45% to 50% under stress condition.^16^ Myocardial perfusion images during stress and rest are compared to detect the stress-induced ischemic change or myocardial injury (Figure 1).^17^,^18^ Several perfusion tracers are used to assess coronary artery disease (CAD) (Table 2, Figure 2).^17^,^19^–^22^

**Table 2:** Tracers for perfusion imaging

Tracer	Chemical structure*	Type of tracer	Production	Half -life	Positron range (mm)	Scan duration (rest and stress)	Intravenously administered activity (MBq)	Effective dose (mSv/MBq)	Approval year		
									FDA	Europe	Japan
SPECT											
^201^Thalium	^201^Tl^+^	Metal cation	Cyclotron	73 h	–	4-h	74–148	0.23	1977	1980**	1991
^99m^Tc-sestamibi		Metal complex	Generator	6 h	–	4-h or 2-days	740–1480	0.0085	1990	1987**	1993
^99m^Tc-tetrofosmin		Metal complex	Generator	6 h	–	4-h or 2 days	740–1480	0.0067	1996	1993**	1996
^99m^Tc-teboroxime			Generator	6 h	–				1990	–	–
PET											
^82^Rubidium	^82^Rb^+^	Metal cation	Generator	76 s	8.6	30-min	370–740	0.0048	1989	–	–
^13^N-ammonia	^13^NH_3_	Inorganic compound	Cyclotron	9.96 min	2.53	1.5-h	370–740	0.0022	2007	–	2012
^15^O-water	H_2_^15^O	Inorganic compound	Cyclotron	2.04 min	4.14	30-min	370–740	0.0011	–	–	–
^18^F-flurpiridaz		Organic compound	Cyclotron	109.8 min	1.03	1.5-h or 2 days	222–370	0.019	–	–	–

*The mass number of ^99m^Tc was omitted

**EURD List Juli 2017: http://www.ema.europa.eu/docs/en_GB/document_library/Other/2012/10/WC500133159.xls

#### SPECT tracers for perfusion imaging

Thallium-201 (^201^Tl), technetium-99m (^99m^Tc)-sestamibi, and ^99m^Tc-tetrofosmin are available for SPECT myocardial perfusion imaging (MPI).

### ^99m^Tc-labeled myocardial perfusion tracers. Thallium-201

^201^Tl, introduced in the 1970 s, was the first SPECT MPI tracer available in a clinical setting.^23^ In 1975, Wackers et al. reported on the imaging of acute myocardial infarction with ^201^Tl.^24^ ^201^Tl is produced in a cyclotron and has a relatively long half-life (73 hours), and therefore requires lower injection doses to minimize radiation exposure. ^201^Tl is a potassium analog and is transported into the myocyte via cell membrane Na^+^/K^+^ pumps during the first transit in proportion to regional MBF.

^201^Tl emits low-energy photons (71 to 80 keV), therefore requiring longer imaging acquisition times and resulting in limited image quality due to absorption and photon scattering especially in obese patients. Biodistribution of ^201^Tl is generally proportional to organ blood flow. Injected ^201^Tl is rapidly cleared from the blood with maximal concentration by normal myocardium (5% to 8% remains in the blood at 5 minutes). The whole-body retention curve can be represented by a biexponential curve. ^201^Tl is excreted slowly in both feces and urine. Approximately 4% to 8% of the administered dose is excreted in the urine in the first 24 hours.^25^,^26^ Lung uptake of ^201^Tl is generally low. An increased lung uptake is known to be associated with greater segmental myocardial perfusion abnormality, increased severity and extent of CAD, and subsequent adverse cardiac events.^27^

Whole-body radiation exposure after an injection (2 to 4 mCi) is up to ~ 25 mSv.^28^,^29^

**Figure 3:**
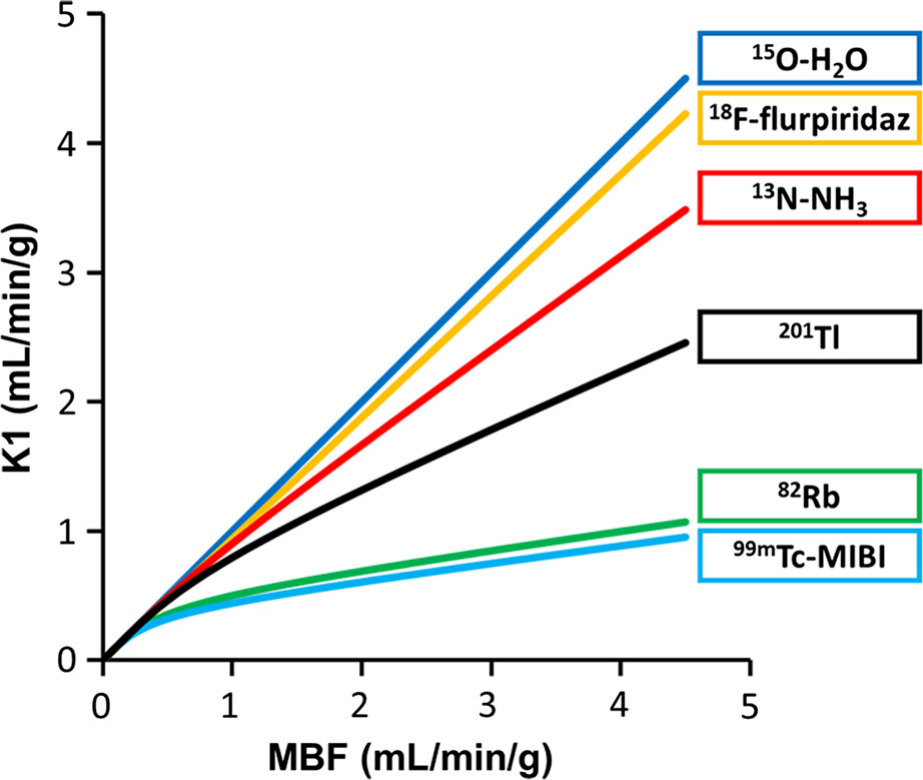
Extraction fraction of each perfusion tracer The extraction fraction of ^15^O-H_2_O is nearly 100% due to its exclusive property of being metabolically inert and freely diffusible. The extraction fraction of ^82^Rb is lower than that of the other PET tracers. ^201^Tl has a higher extraction fraction compared to that associated with ^99m^Tc-MIBI

^201^Tl has a higher extraction coefficient than do ^99m^Tc-labeled perfusion tracers (Figure 3). The higher extraction fraction may be an advantage for MBF quantification.^30^

Stress images are acquired 5 to 15 minutes after tracer injection in order to avoid the “upward creep” phenomenon due to rapid respiration if the stress is produced through exercise. Redistribution images are acquired 2 to 4 hours after initial injection. Differential washout rates of normal regions (with faster washout) vs regions with ischemic segments (slower washout) contribute to the redistribution or normalization of the abnormal regions in delayed images.

#### ^99m^Tc-labeled myocardial perfusion tracers

^99m^Tc is a generator-produced agent eluted from molybdenum-99 (^99^Mo). Despite its initial Food and Drug Administration (FDA) approval, ^99m^Tc-teboroxime is far less commonly used due to the excessive initial uptake in the myocardium and rapid washout.^31^,^32^ ^99m^Tc-sestamibi and ^99m^Tc-tetrofosmin have had widespread clinical use. The first use of ^99m^Tc-tetrofosmin for humans was reported in 1993 as part of a phase 1 clinical trial.^33^ Injected ^99m^Tc-labeled perfusion tracer distributes in the myocardium according to regional myocardial perfusion. Its uptake by myocardium is related to the presence of intact mitochondria.^34^

Because its half-life is 6 hours, the administered dose is relatively larger and the radiation exposure is lower respectively than those associated with ^201^Tl.^29^ The peak energy level of γ-rays from ^99m^Tc is about 140 keV, which is suitable for γ-camera imaging and electrocardiographically (ECG) gated myocardial perfusion SPECT.

^99m^Tc-sestamibi is rapidly cleared from blood after intravenous administration. Lung uptake is generally low. However, marked accumulation is present in liver and spleen at resting condition during the first 60 minutes after injection. After an injection with exercise stress, substantially less uptake is observed in the liver and spleen with excellent visualization of heart.^35^ ^99m^Tc-tetrofosmin is rapidly cleared from the blood (< 5% remains in blood by 10 minutes) after intravenous administration. Uptake in myocardium is approximately 1.2% with minimal redistribution, and approximately 1% at 2 hours. Clearance from liver is quick (< 4.5% remains by 60 minutes) and lung uptake is also rapidly reduced.^33^,^36^,^37^ Myocardial uptake of ^99m^Tc-tetrofosmin is higher from 5 to 60 minutes than is that for ^99m^Tc-sestamibi. The biological half-life of ^99m^Tc-tetrofosmin in normal myocardium and liver is significantly shorter than that of ^99m^Tc-sestamibi. Heart-to-lung ratios for ^99m^Tc-tetrofosmin and ^99m^Tc-sestamibi are similar, whereas heart-to-liver ratios for ^99m^Tc-tetrofosmin are significantly higher from 30 to 60 minutes post injection compared to those for ^99m^Tc-sestamibi.^37^,^38^

Total whole-body radiation after a typical injection dose (10 to 25 mCi) is ~ 10.6 mSv for ^99m^Tc-tetrofosmin and 12.0 mSv for ^99m^Tc-sestamibi.^28^

Separate stress and rest injections are required for the detection of stress-induced ischemia due to its slow clearance from myocytes. Both ^99m^Tc-sestamibi and ^99m^Tc-tetrofosmin have lower extraction coefficients than does ^201^Tl (Figure 3).^39^ Recent SPECT systems allow the quantification of MBF from dynamic tracer imaging due to the improved sensitivity and temporal resolution.^40^,^41^

#### PET tracers for myocardial perfusion imaging

**Figure 4:**
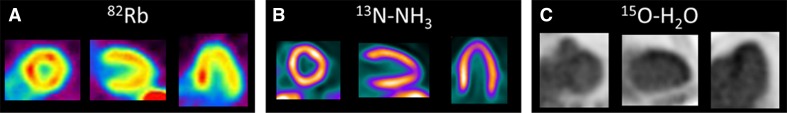
Qualitative images of PET tracers ^82^Rb PET has relatively low lesion contrast with low spatial resolution. ^13^N-NH_3_ PET shows clear images due to rapid clearance from the blood pool. With ^15^O-H_2_O PET, it is difficult to distinguish between myocardium and blood pool

Several PET tracers can be used to assess myocardial perfusion.^18^ These include ^82^Rb, ^13^N-NH_3_, and ^15^O-H_2_O (Figure 4).^19^ Both ^13^N-NH_3_ and ^82^Rb are commonly used for both qualitative and quantitative measurements.^34^,^42^–^44^ Visual assessment of PET myocardial perfusion imaging provides high diagnostic accuracy in the detection of CAD.^17^ Dynamic imaging analysis permits quantitative assessment of MBF and coronary flow reserve (CFR), which is defined as the ratio of MBF at peak hyperemia to MBF at rest. CFR measurements provide additional value in the detection of multi-vessel disease and risk stratification of CAD patients.^45^–^49^ ^15^O-H_2_O is an ideal myocardial flow tracer to quantify MBF with a linear relation between first-pass extraction and perfusion, but the perfusion images are not of high quality as they are with the other 2 PET tracers (Figure 3).^19^,^30^,^50^,^51^

^82^Rb is the most widely used tracer because it is a strontium-82 (^82^Sr)/^82^Rb generator-produced tracer that does not require a cyclotron for its production.^52^,^53^ Love et al. initially developed rubidium-86 for myocardial perfusion imaging with a dog.^7^ Following non-human studies, Selwyn et al. applied ^82^Rb to a human for the first time in 1982.^54^. The short physical half-life of ^82^Rb (76 seconds) enables sequential rest/stress scanning. ^82^Rb is a potassium analog, and therefore injected ^82^Rb is actively transported into myocytes through the Na^+^/K^+^ adenosine triphosphate (ATP) transport system. This uptake of ^82^Rb is dependent on MBF and its first-pass retention fraction is approximately 65% at rest. The relatively low lesion contrast with low spatial resolution due to the lower extraction fraction and high positron range is a slight disadvantage of ^82^Rb.^39^ In 2000, ^13^N-NH_3_ PET was approved by the United States Food and Drug Administration (FDA) to evaluate myocardial perfusion in patients with known or suspected CAD.^19^ ^13^N-NH_3_ was also approved by the Japanese Ministry of Health and Welfare in March 2012 (Table 2).^55^

The uptake mechanism of ^13^N-NH_3_ is unclear. After being taken into the myocyte, ^13^N-NH_3_ underwent metabolic trapping with the conversion of NH_3_ to glutamine, glutamic acid, and carbamoyl phosphate.^56^ ^13^N-NH_3_ PET is suitable for imaging and measuring of MBF due to its high first-pass extraction fraction and retention in the myocardium with rapid clearance from the blood pool, which also give it high diagnostic accuracy.^57^ The requirement for a cyclotron limits the clinical use of ^13^N-NH_3_. Its relatively longer half-life (9.96 minutes) necessitates a longer interval between rest and stress scans, resulting in low throughput in a clinical setting. These are the main disadvantages of ^13^N-NH_3_.^39^ The FDA has approved ^82^Rb and ^13^N-NH_3_ for clinical use (Table 2). The Japanese Ministry of Health, Labour, and Welfare has approved ^13^N-NH_3_ for detecting CAD in cases of CAD unable to be diagnosed with using SPECT MPI.^55^

^15^O-H_2_O is unique in being metabolically inert and freely diffusible, which are considered ideals for measuring MBF due to the linear relationship between first-pass extraction and perfusion.^58^ The shorter half-life (2.04 minutes) enables consecutive rest/stress protocols, similar to the case with ^82^Rb.^59^,^60^ However, ^15^O-H_2_O requires an on-site cyclotron for tracer production and also is suboptimal for visual assessment due to the low signal-to-noise ratios. These conditions lead to its use being limited in clinical settings. ^15^O-H_2_O has gained wide popularity in research settings due to its excellent kinetic properties.^19^,^61^–^63^ A recent study by Danad et al. examined stress MBF and CFR in 330 patients with CAD,^64^ possibly indicating that ^15^O-H_2_O could move from research to clinical use.

Fluorine-18 (^18^F)-flurpiridaz, an analog of the insecticide pyridaben, is a novel MPI tracer that can bind to the mitochondrial complex-1 inhibitor.^51^,^65^ The positron range of ^18^F is 1.03 mm, shorter than that of other PET perfusion tracers (Table 2). Injected ^18^F-flurpiridaz shows very high first-pass extraction and high affinity in myocardial tissue with slow washout from cardiomyocytes (Figure 3). Therefore, accurate quantification of MBF and CFR measurements with high image quality and excellent diagnostic accuracy are expected.^66^–^68^ Because of the longer half-life of ^18^F (109.8 minutes), delivery of unit doses from regional cyclotrons may be possible, similar to the case with fluorine-18-labeled fluorodeoxyglucose (^18^F-FDG). In the meantime, repeated measurements of stress and rest studies would likely be difficult due to the longer half-life, and therefore a separate day protocol or some correction for the residual activity of the first acquisition might be needed. Phase 2 clinical trials showed promise,^67^ and phase 3 clinical trials demonstrated the diagnostic usefulness for specific subpopulations such as women and obese patients.

### Metabolic Imaging

**Figure 5:**
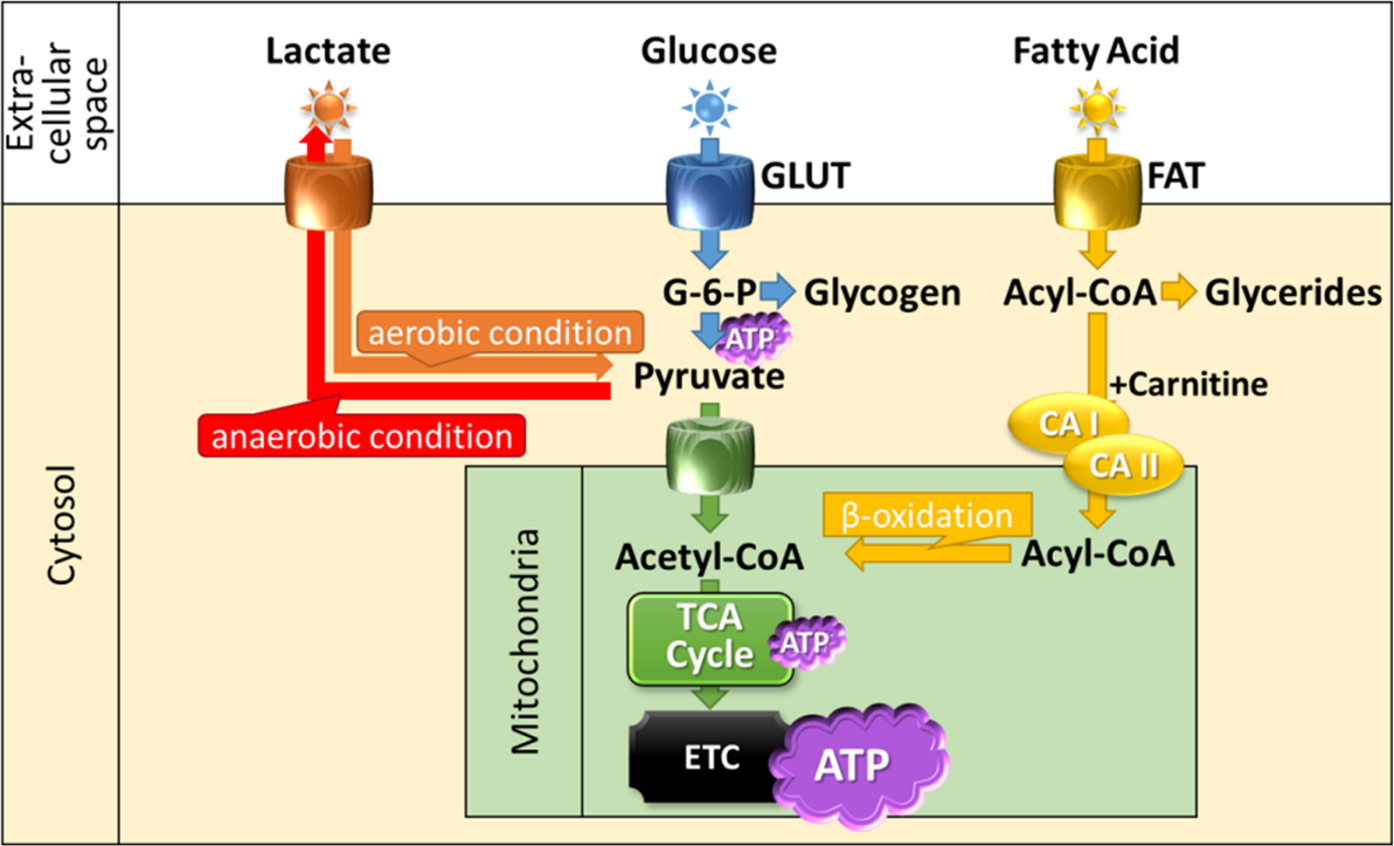
Schematic representation of cardiac energy metabolism Substrates are transported across the extracellular membrane into the cytosol through GLUT for glucose and FAT for fatty acid. Metabolized intermediates such as pyruvate and acyl-CoA are transported across the inner mitochondrial membrane for oxidation. Then inside the mitochondrion, substrates are oxidized or carboxylated and fed into the TCA cycle and ETC to produce ATP. *GLUT*, glucose transporter; *FAT*, fatty acid transporter; *G-6-P*, glucose-6-phosphate; *ATP*, adenosine triphosphate; *TCA*, tricarboxylic acid; *ETC*, electron transport chain; *CA I*, carnitine acyltransferase I; *CA II*, carnitine acyltransferase II

**Figure 6:**
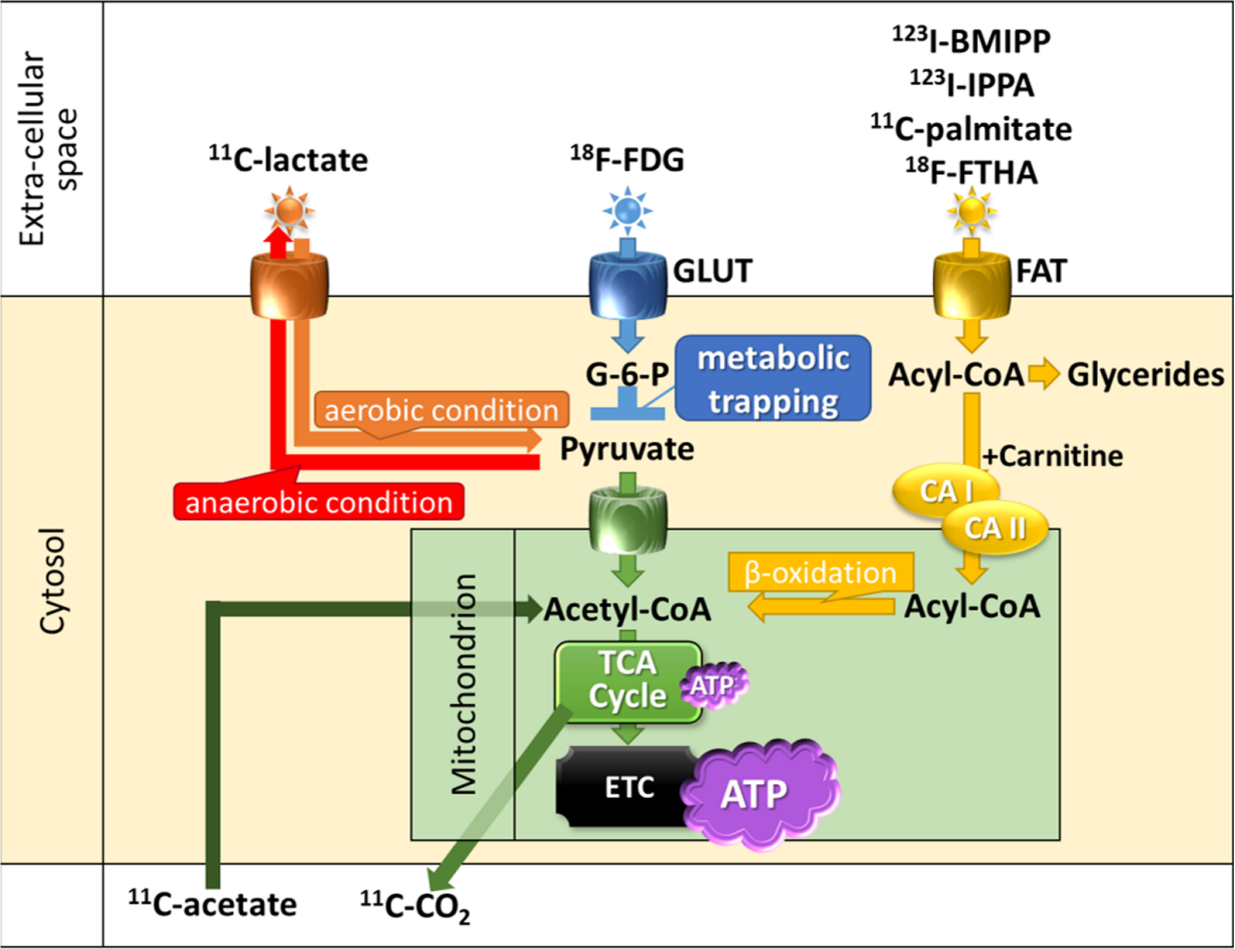
Tracers for assessing cardiac energy metabolism ^18^F-FDG is a glucose analog in which the oxygen in position C-2 is replaced with ^18^F. ^18^F-FDG is actively transported into the cell mediated by GLUT in the same way as glucose. Once inside the cell, glucose and ^18^F-FDG are phosphorylated by hexokinase. Phosphorylated glucose (G-6-P) continues along the glycolytic pathway for energy production. However, ^18^F-FDG-6-phosphate cannot enter glycolysis and is trapped intracellularly in a condition known as “metabolic trapping.” *GLUT*, glucose transporter; *G-6-P*, glucose-6-phosphate; *FDG*, ^18^F-fluorodeoxyglucose; *FDG-6-P*, ^18^F-FDG-6-phosphate

The heart derives its energy from a variety of sources such as free fatty acids (FFA), glucose, lactate, and ketone bodies (Figure 5).^69^ Glucose metabolism dominates after feeding, and fatty-acid metabolism dominates under long-fasting conditions.^69^ Carbohydrates taken into cardiomyocytes are metabolized into pyruvic acid using various enzymatic actions. If oxygen supply is sufficient, ATPs are produced from glucose via the glycolysis system in the tricarboxylic acid (TCA) cycle and electron transfer system.^70^ In the ischemic state, acid metabolism is impaired due to insufficient oxygen supply to the myocardium.^71^ Alternatively ATP is produced from lactic acid because anaerobic glycolysis with less oxygen consumption becomes predominant. However, anaerobic glycolysis produces less ATP than does aerobic glycolysis. If severe myocardial ischemia continues, myocardial cells become necrotic as ATP production diminishes.^72^ Several SPECT and PET tracers have been used or tried clinically to assess myocardial metabolism (Table 3, Figure 6).

**Table 3:** Tracers for metabolic imaging

Tracer	Chemical structure	Characteristics	Approval year		
			FDA	Europe	Japan
SPECT					
^123^I-BMIPP		Long-chain fatty acid analogue	–	–	1993
^123^I *p*-IPPA		Long-chain fatty acid analogue	–	–	–
^123^I *o*-IPPA		Long-chain fatty acid analogue	–	–	–
^123^I-9-MPA		Long-chain fatty acid analogue	–	–	–
PET					
^18^F-FDG		Glucose analog	1997	1994*	2002
^18^F-FTHA		Long-chain fatty acid analog	–	–	–
^11^C-palmitatic acid		Long-chain fatty acid analog	–	–	–
^11^C-acetic acid		^11^C labeled acetic acid, oxidative metabolism	–	–	–

*BMIPP*, beta-methyl-p-iodophenylpentadecanoic acid; *IPPA*, iodophenylpentadecanoic acid; *9-MPA*, iodophenyl-9-methyl-pentadecanoic acid; *FDG*, fluorodeoxyglucose; *FTHA*, fluoro-6-thia-heptadecanoic acid

*EURD List Juli 2017: http://www.ema.europa.eu/docs/en_GB/document_library/Other/2012/10/WC500133159.xls

#### SPECT tracers for metabolic imaging

For fatty acid metabolism evaluation, SPECT examination using iodine-123-labeled beta-methyl-*p*-iodophenylpentadecanoic acid (^123^I-BMIPP) has been clinically used in Japan.^44^,^73^,^74^ However, ^123^I-BMIPP was initially developed in the United States and the first human use was in 1986 by Knapp et al.^75^ After the initial development in the US, the Japanese community took over development of ^123^I-BMIPP. The first human use in Japan was reported in 1991 in a Japanese article.^76^ Following this Japanese article, Kurata et al. reported Japanese ^123^I-BMIPP data in an international journal in 1992.^77^ ^123^I-BMIPP is an iodinated fatty-acid analog used to assess myocardial fatty acid metabolism.^78^,^79^ This tracer, however, is not approved for clinical use in the US despite its successful for clinical use even successive early experience use in that country.^80^ Iodine-123-labeled iodophenylpentadecanoic acid (^123^I-IPPA) is a radiolabeled free fatty acid (FFA) analog which is in phase 3 trials in United States but which has not yet been approved.^81^

Following intravenous injection, ^123^I-BMIPP and ^123^I-IPPA are rapidly distributed to various organs, such as liver and heart, and cleared rapidly from the blood.^81^–^84^ Initial uptake of the administered dose of ^123^I-BMIPP is assumed to be about 6% by the heart and 14% by the liver. The residual ^123^I-BMIPP is distributed uniformly in other organs and tissues.^76^,^85^,^86^ After initial uptake, only a portion of the ^123^I-BMIPP and ^123^I-IPPA is metabolized immediately to water-soluble low-molecular-weight products. Most of the ^123^I-IPPA undergoes metabolism similar to that of long-chain fatty acids, through rapid mitochondrial beta-oxidation.^87^,^88^ The initial and late clearance of ^123^I-IPPA are thought to reflect β-oxidation and clearance of tracer incorporated into triglyceride pools, respectively.^88^ ^123^I-IPPA images show minimal background activity and good image quality. The metabolism of ^123^I-BMIPP is slower than that of ^123^I-IPPA because ^123^I-BMIPP is a modified-branched fatty acid analog with a methyl group on the beta-carbon. Both of the end products are excreted in a conjugated form in the urine.^76^,^89^,^90^

**Figure 7:**
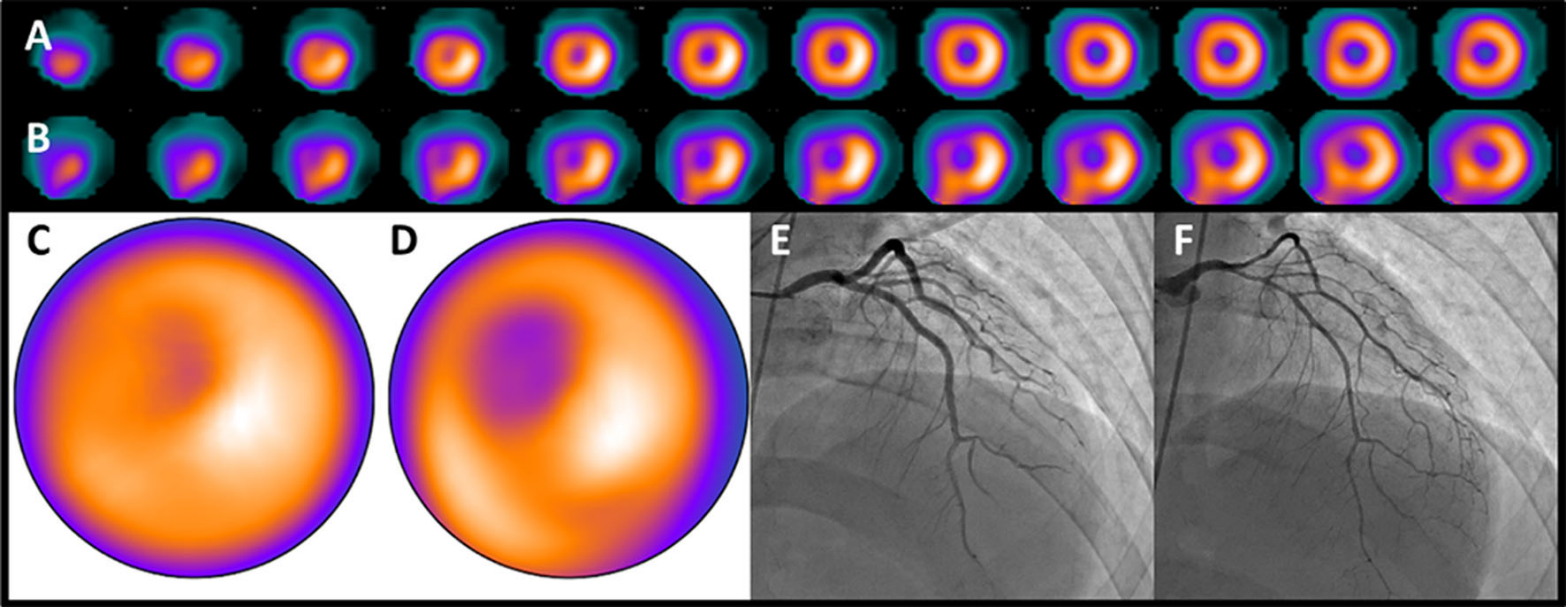
Ischemic memory imaging Perfusion image of ^99m^Tc product shows slightly reduced perfusion (**A**, **C**), whereas moderately reduced ^123^I-BMIPP uptake is seen in the anterior to septal wall (**B**, **D**), which indicates perfusion-metabolic mismatch. Coronary angiogram shows no significant stenosis (**E**); however, vasospastic angina in the left anterior descending artery due to the spasm is proved through intracoronary injection of acetylcholine (**F**)

^123^I-BMIPP scintigraphy when combined with perfusion imaging may show preserved perfusion, but fatty acid metabolism is impaired as myocardium shifts from metabolizing fatty acids to metabolizing predominantly glucose following ischemic episodes. Therefore, the region of perfusion-metabolic mismatch (^123^I-BMIPP defect larger than perfusion defect) indicates the presence of ischemic myocardium (Figure 7). 80,91–93 ^123^I-BMIPP has been approved in Japan only for clinical use.^44^

#### PET tracers for metabolic imaging

^18^F-FDG is the most frequently used tracer around the world and is employed mainly for the assessment of malignant tumors. For the purposes of nuclear cardiology imaging, ^18^F-FDG PET was first used to define and identify viable myocardium in CAD in the 1980 s.^94^ Since ^18^F-FDG is an analog of glucose, once taken up into the cardiomyocytes via the glucose transporter (GLUT), it is phosphorylated to ^18^F-FDG-6-phosphate by hexokinase as well as glucose.^95^ ^18^F-FDG-6-phosphate accumulates intracellularly without being metabolized during glycolysis, a condition referred to as “metabolic trapping” (Figure 6). Therefore, myocardial viability can be evaluated by assessing the accumulation of ^18^F-FDG in myocardium. To determine myocardial viability, oral glucose loading or an insulin-glucose clamp is applied to enhance ^18^F-FDG uptake in viable myocardium.^96^,^97^ In ischemic myocardium, ^18^F-FDG accumulation in the myocardium is maintained under a fasting condition due to the dominant anaerobic glucose metabolism. On the other hand, in the infarcted scar tissue, ^18^F-FDG accumulation is absent due to non-availability of glucose metabolism. In a clinical setting, ^18^F-FDG PET viability assessment is performed using the myocardial perfusion image obtained by SPECT or PET.^94^,^98^ A region with preserved ^18^F-FDG accumulation but reduced myocardial perfusion indicates viable myocardium. In such a case, functional recovery after coronary revascularization is likely especially with extensive mismatch pattern.

^11^C-palmitate and fluorine-18-labeled fluoro-6-thia-heptadecanoic acid (^18^F-FTHA) have been used to evaluate fatty acid metabolism.^99^–^101^ Similar to the case with to ^18^F-FDG PET, a shift in myocardial metabolism from fatty acid to glucose can be estimated using these fatty acid analogs.^102^

Myocardial oxygen metabolism can be non-invasively evaluated by ^11^C-acetate PET.^103^,^104^ ^11^C-acetate taken into myocardium is converted into acetyl-CoA, consecutively metabolized and excreted into ^11^C-CO_2_ via the TCA cycle. The ^11^C-acetate clearance rate is used to assess myocardial oxygen consumption since TCA cycle activity is directly linked with myocardial oxygen consumption which is independent of the concentration of energy substrates for the myocardium.^105^,^106^ ^11^C-acetate PET allows for non-invasive observation of regional myocardial oxygen metabolism in the presence of ischemia,^107^,^108^ cardiomyopathy,^109^,^110^ and heart failure (HF) in a state of deprived energy.^111^,^112^ Myocardial oxidative metabolism in the RV can also be estimated using ^11^C-acetate PET.^113^–^116^ ^11^C-acetate PET permits the evaluation of both blood flow and oxygen metabolism with one examination using some model analysis due to the relatively high extraction fraction.^62^

### Sympathetic Imaging

**Figure 8:**
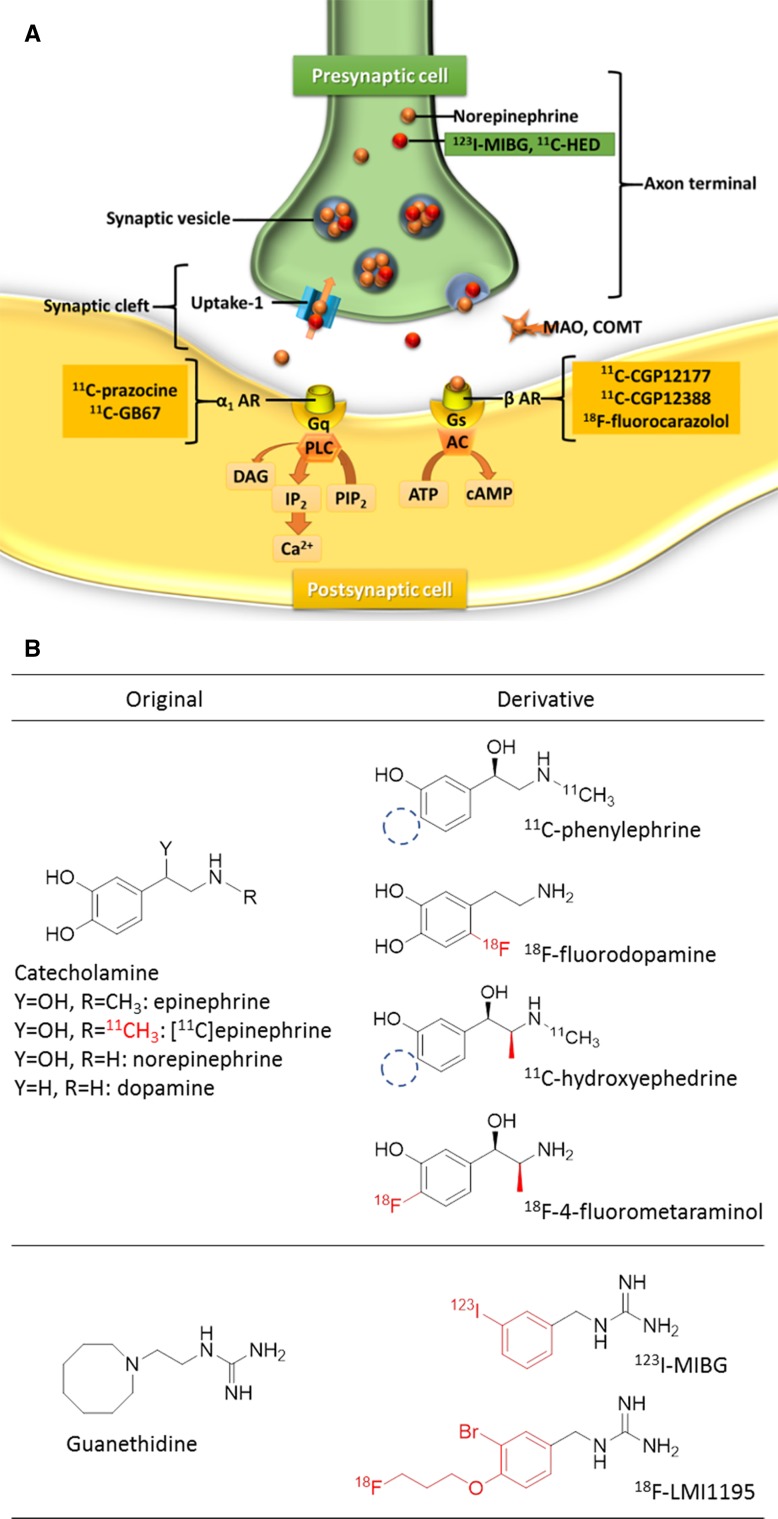
Schema of myocardial adrenergic neuronal terminals Figure **A** shows the schematic representation of myocardial adrenergic neuronal terminals and Figure **B** shows the chemical structure of each tracer. MIBG is actively taken up into sympathetic nerves through the uptake-1 mechanism and then stored in the synaptic vesicle in a manner similar to that for norepinephrine (NE). Nerve stimulation releases MIBG and NE into the synaptic cleft through exocytosis. MIBG does not bind to the postsynaptic receptor and is not metabolized by monoamine oxidase (MAO) or catechol-O-methyltransferase (COMT). Most of the released MIBG undergoes reuptake through the uptake-1 mechanism, and the remaining MIBG goes into the blood (spillover). ^*123*^ *I-MIBG*, *m*-[^123^I]iodobenzylguanidine; ^*11*^ *C-HED*, ^11^C-hydroxyephedrine; *DAG*, diacylglycerol; *AR, adrenergic receptor*;*Gq*, phospholipase C-coupled Gq-protein; *Gs*, phospholipase C-coupled Gs-protein; *ATP*, adenosine triphosphate; *cAMP*, cyclic adenosine monophosphate; *IP*_*2*_, inositol bisphosphate; *PIP*_*2*_, phosphatidylinositol biphosphate

The heart has extensive innervation, both sympathetic and parasympathetic. The sympathetic nervous system uses norepinephrine (NE), and the parasympathetic nervous system uses acetylcholine (Ach) as the main neurotransmitters. NE is synthesized from the amino acid tyrosine in presynaptic neurons (Figure 8). NE is transported into the presynaptic neuronal terminal vesicles by the vesicular monoamine transporter (VMAT). Exocytosis is led by the activation of voltage-dependent calcium channels and vesicles at the presynaptic neuron. Some of the NE released into the synaptic cleft binds to the adreno-receptors for downstream effects, while much of the NE undergoes reuptake into presynaptic neurons via the terminal transporter (uptake-1).^117^–^119^

The sympathetic nerve is vulnerable to ischemia, and sympathetic nervous function may decline even if myocardial blood flow at rest is maintained.^120^ In HF, continued stimulation of the β1 receptor due to increased norepinephrine levels results in a decrease of receptor density (down regulation), with corresponding poor reactivity to the stimulation. Moreover, in a persistent state of sympathetic hyperactivity, the ability to retain norepinephrine is also decreased at the nerve terminal end.^121^ Abnormal neuro-hormonal function is reported in various heart diseases, and worsening of neuronal function is associated with cardiac events and sudden cardiac death.^122^–^124^

#### SPECT tracers for sympathetic imaging

**Figure 9:**
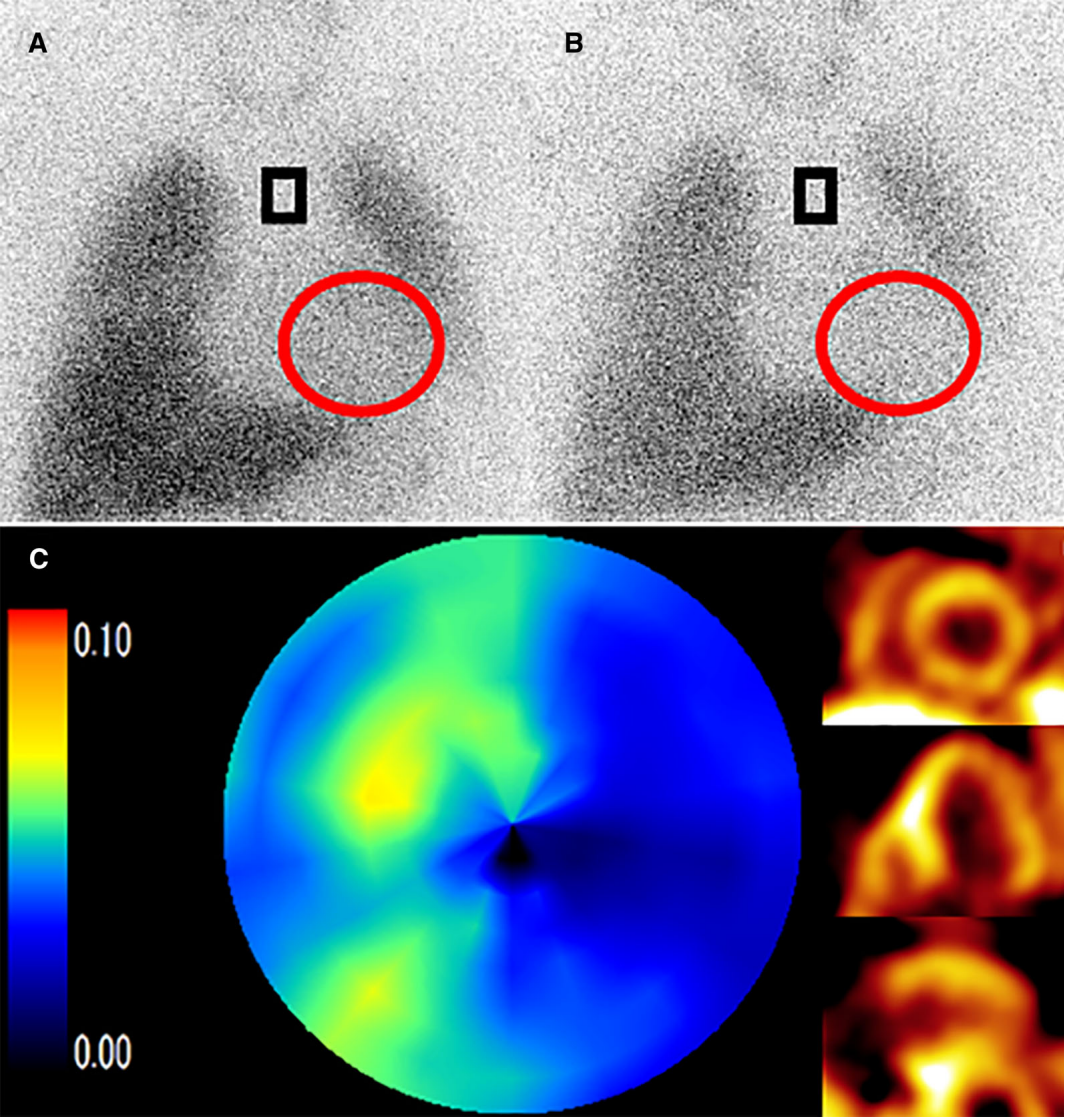
Representative case of ^123^I-MIBG scintigraphy and ^11^C-hydroxyephedrine PET A male in his 40s suffered from dilated cardiomyopathy, with a left ventricular ejection fraction of approximately 30%. An early anterior planar image at 15 min after injection (**A**) and a late anterior planar image starting at 4 hours after injection (**B**) are acquired to calculate the heart-to-mediastinum ratio (HMR) and the washout ratio. Calculated early HMR, delayed HMR, and washout ratio were 1.7, 1.4, and 40.3%, respectively. Whole retention index from ^11^C-hydroxyephedrine PET was calculated as 0.044. Distribution of sympathetic nerve system was lower especially in the lateral wall

Iodine-123-labeled metaiodobenzylguanidine (^123^I-MIBG) is widely used as a SPECT tracer to evaluate the presynaptic sympathetic innervation of the heart.^125^–^127^ The first use of ^123^I-MIBG in humans was in 1981 by a University of Michigan group.^128^ It is an analog of catecholamine, which is taken up via the uptake-1 mechanism and stored in synaptic vesicles as is NE. Tracers are released into the synaptic cleft from the synaptic vesicle via the exocytosis pathway, but do not lead to any physiological activity without binding to the catecholamine receptor. Since it is not metabolized by monoamine oxidase (MAO) or catechol-O-methyltransferase (COMT), most of the released tracer is reabsorbed at the synapse terminal and again stored in synaptic vesicles. Therefore, information reflecting the process of ^123^I-MIBG uptake into the synapse terminal, storage in the vesicles, secretion, reabsorption, and release into the blood is obtained from sympathetic imaging.^129^,^130^ An early anterior planar image at 15 minutes after injection and a late anterior planar image starting at 3 to 4 hours after injection are acquired to calculate the heart-to-mediastinum ratio (HMR) and the washout ratio (Figure 9). These parameters are considered to be standards. The high liver uptake and relatively high energy of the tracers make the image quality suboptimal. It is difficult to evaluate SPECT images especially in severe HF, which usually has limited myocardial ^123^I-MIBG radioactivity. Therefore, planar data acquisition is standard for ^123^I-MIBG imaging.^131^ Although these images present an easily obtained index, inter-institutional differences of the HMR due to differences in camera-collimator systems being used have hampered multicenter comparisons. Recently, standardization among different collimator types has been achieved using the calibration phantom and could easily be extrapolated to the images of other institutions.^132^,^133^ Late HMR provides the relative distribution of cardiac sympathetic nerve terminals, which is related to neuronal function from uptake to release. Washout ratio represents the information of the sympathetic drive. Several studies have presented that patients with chronic HF and a low late HMR and/or an increased washout rate are at increased risk for cardiac death.

#### PET tracers for sympathetic imaging

As a PET tracer, carbon-11-labeled hydroxyephedrine (^11^C-HED) is used mainly to assess presynaptic cardiac sympathetic nerve distribution.^134^ ^11^C-HED is still the most widely used PET tracer for sympathetic nervous function imaging in mainly research settings.^135^ Extracardiac uptake is mainly by the liver with very limited lung uptake. In ischemic heart disease, a mismatch region of myocardial blood flow and sympathetic dysfunction is reported as a decision criterion for prediction of fatal arrhythmia and indication for cardioverter-defibrillator implantation (ICD).^136^,^137^ The distribution abnormality of cardiac sympathetic denervation has been demonstrated in previous ^11^C-HED studies, including those involving patients with HF,^138^,^139^ cardiac arrhythmias,^140^,^141^ myocardial infarction,^142^,^143^ cardiac diabetic neuropathy,^144^,^145^ and HF with preserved ejection fraction (HFpEF).^146^

N-[3-bromo-4-(3-^18^F-fluoro-propoxy)-benzyl]-guanidine (LMI 1195) is a novel ^18^F-labeled ligand to image the norepinephrine transporter.^147^ ^18^F-fluorometaraminol,^148^ ^11^C-phenylephrine,^149^ ^18^F-fluorodopamine,^150^ and ^11^C-epinephrine^151^ are the other radiotracers for evaluating presynaptic neuronal function. Several tracers such as ^18^F-fluorocarazolol,^152^ 4-[3-[(1,1-dimethyl)amino]-2-hydroxypropoxy]-1,3-dihydro-2H-benzimidazol-2-^11^C-one (^11^C-CGP12177),^153^ and (S)-4-(3-(2’-^11^C-isopropylamino)-2-hydroxypropoxy)-2H-benzimidazol-2-one (^11^C-CGP12388)^154^ have been reported for assessing postsynaptic sympathetic neuronal functions through measurement of myocardial β-adrenergic receptor (β-AR) density, which directly regulates LV systolic function.^155^ There are several reports regarding tracers for imaging the parasympathetic nervous system,^156^,^157^ but the clinical role of these has not yet been established (Table 4).

**Table 4:** Tracers for sympathetic imaging

			Tracer	Chemical structure	Approval year		
					FDA	Europe	Japan
Sympathetic nervous system							
Presynaptic							
Catecholamine derivative			^123^I-MIBG		2008	1995**	1992
			^11^C-hydroxyephedrine		–	–	–
			^18^F-LMI1195		–	–	–
			^18^F-4-fluorometaraminol (4-[^18^F]fluorometaraminol)		–	–	–
Catecholamine			^18^F-fluorodopamine		–	–	–
			^11^C-epinephrine		–	–	–
			^11^C-phenylephrine		–	–	–
Postsynaptic							
α-receptor			^11^C-prazosin		–	–	–
β-receptor			^11^C-CGP12177		–	–	–
			^11^C-CGP12388		–	–	–
			^18^F-fluorocarazolol		–	–	–
Parasympathetic nervous system							
Presynaptic		Vesicular acetylcholine transporter	^18^F-fluoroethoxybenzovesamicol		–	–	–
Postsynaptic		Muscarine	^11^C-methyl QNB		–	–	–
			^11^C-methyl TRB		–	–	–

**EURD list nov 2012 http://www.ema.europa.eu/docs/en_GB/document_library/Other/2012/04/WC500124999.xls

### Imaging of Inflammation and Atherosclerosis

Nuclear medicine imaging can be used to view several in vivo pathological processes in inflammation and atherosclerosis. Several novel tracers may have uses for tracking inflammation, hypoxia, or active calcification (Table 5).

**Table 5:** Inflammation and atherosclerosis imaging

Isotope	Radiopharmaceutical		Type of tracer	Study population	Characteristics	Approval year		
						FDA	Europe	Japan
General inflammation								
^18^F	^18^F-FDG		Organic compound	Carotid and coronary plaque imaging Cardiac sarcoidosis Device infection	Accumulating macrophage Strong signal Limitation: non-specific myocardial accumulation	–	1994*	2012 (cardiac sarcoidosis)
^67^Ga	^67^Gallium	^67^Ga^3+^	Metal cation	Inflammatory heart disease Cardiac sarcoidosis	No physiological uptake Limitation: suboptimal image quality	1976	1972*	1982
Infection								
^111^In	^111^In WBC		Radiolabeled cell	Infectious disease	Accumulates in WBC Limitation: suboptimal image quality	1985	1980**	1992
Atherosclerosis imaging								
^99m^Tc	^99m^Tc annexin 5		Radiometal-tagged Annexin V	Apoptosis imaging	Lesion specific Limitation: weak signal intensity	–	–	–
^68^Ga	^68^Ga DOTATATE		Radiometal-tagged octreotide analog	Symptomatic carotid atherosclerosis Unstable angina	Accumulates activated macrophages No physiological myocardial uptake Generator produced	2016	–	–
^64^Cu	^64^Cu DOTATATE		Radiometal-tagged octreotide analog	Symptomatic carotid atherosclerosis	Good image quality	–	–	–
Translocator protein								
^11^C	^11^C-PK11195		Organic compound	Symptomatic carotid atherosclerosis	Accumulates in activated mononuclear phagocyte	–	–	–
^18^F	^18^F-FEDAC		Organic compound		Accumulates in activated mononuclear phagocyte High affinity and better image quality	–	–	–
^18^F	^18^F-NaF		Inorganic anion	Aortic stenosis Coronary artery disease Carotid artery plaque	Accumulates in calcification lesion	2012	–	–

^*68*^ *Ga DOTATATE*, Gallium-68-labeled [1,4,7,10-tetraazacyclododecane-*N*,*N*’,*N*’’,*N*’’’-tetraacetic acid]-d-Phe^1^, Tyr^3^-octreotate; ^*18*^ *F-FDG*, ^18^F-fluorodeoxyglucose; ^*18*^ *F-FEDAC*, *N*-benzyl-*N*-methyl-2-[7,8-dihydro-7-(2-[^18^F]fluoroethyl)-8-oxo-2-phenyl-9*H*-purin-9-yl]acetamide; *WBC*, white blood cell

*EURD List Juli 2017: http://www.ema.europa.eu/docs/en_GB/document_library/Other/2012/10/WC500133159.xls

**EURD list 2012 http://www.ema.europa.eu/docs/en_GB/document_library/Other/2012/04/WC500124999.xls

#### SPECT tracers for imaging of inflammation and atherosclerosis

Gallium-67 (^67^Ga) scintigraphy has been used to detect inflammatory lesions including infection and sarcoidosis.^158^,^159^ Several factors influence ^67^Ga accumulation in inflammatory lesions. These factors include increased delivery and accumulation of transferrin-bound ^67^Ga due to increased blood flow and vascular membrane permeability. The tendency of ^67^Ga to bind to lactoferrin and leukocytes also leads to highly concentrated uptake of ^67^Ga.^160^ Imaging is performed at 48 to 72 hours after tracer injection. In clinical settings, physicians ideally look to have results immediately following a diagnostic test, and therefore a late imaging protocol is one of the major limitations of ^67^Ga. ^67^Ga scanning is useful to differentiate acute myocarditis from acute myocardial infarction.^161^ ^67^Ga scintigraphy has been a major analytical tool in the diagnosis of cardiac sarcoidosis.^162^ There is no significant distribution in normal myocardium.^163^ This is an advantage of ^67^Ga when applied to cardiac sarcoidosis. However, generally speaking, ^67^Ga has a limited role in the evaluation and management of sarcoidosis.^163^

Inflammatory cells such as granulocytes, lymphocytes, and macrophages are migrated into inflammatory lesions, resulting in the activation of a biological defense mechanism. SPECT imaging with indium-111 (^111^In)-radiolabeled autologous white blood cells (WBC) has proven to be valuable in the detection of endocarditis. ^111^In-WBC is highly specific for infectious lesions because granulocytes are recruited to the site of inflammatory foci but have limited sensitivity due to a weak signal.^164^^–^ 166

#### Apoptosis imaging

Tissue apoptosis is considered to be one of the earlier stages of vascular plaque rupture,^167^ and therefore detecting apoptotic lesions may precipitate effective treatments to prevent cardiovascular events. Apoptotic cells externalize negatively charged phosphatidylserine (PS).^15^ Human protein annexin A5 binds to PS. ^99m^Tc-labeled annexin A5 has been shown to have higher uptake in the carotid arteries of vulnerable stroke patients.^168^ ^99m^Tc-tagged annexin A5 specifically accumulates in vascular atherosclerotic lesions, which is a great advantage. In contrast, the signal intensity of ^99m^Tc-labeled annexin A5 is quite a bit lower than that of ^18^F-FDG.^169^ ^99m^Tc-labeled annexin A5 drew much interest a decade ago but has not had wide clinical application, perhaps due to the lower signal intensity and tracer availability.

#### PET tracers for imaging of inflammation and atherosclerosis

**Figure 10:**
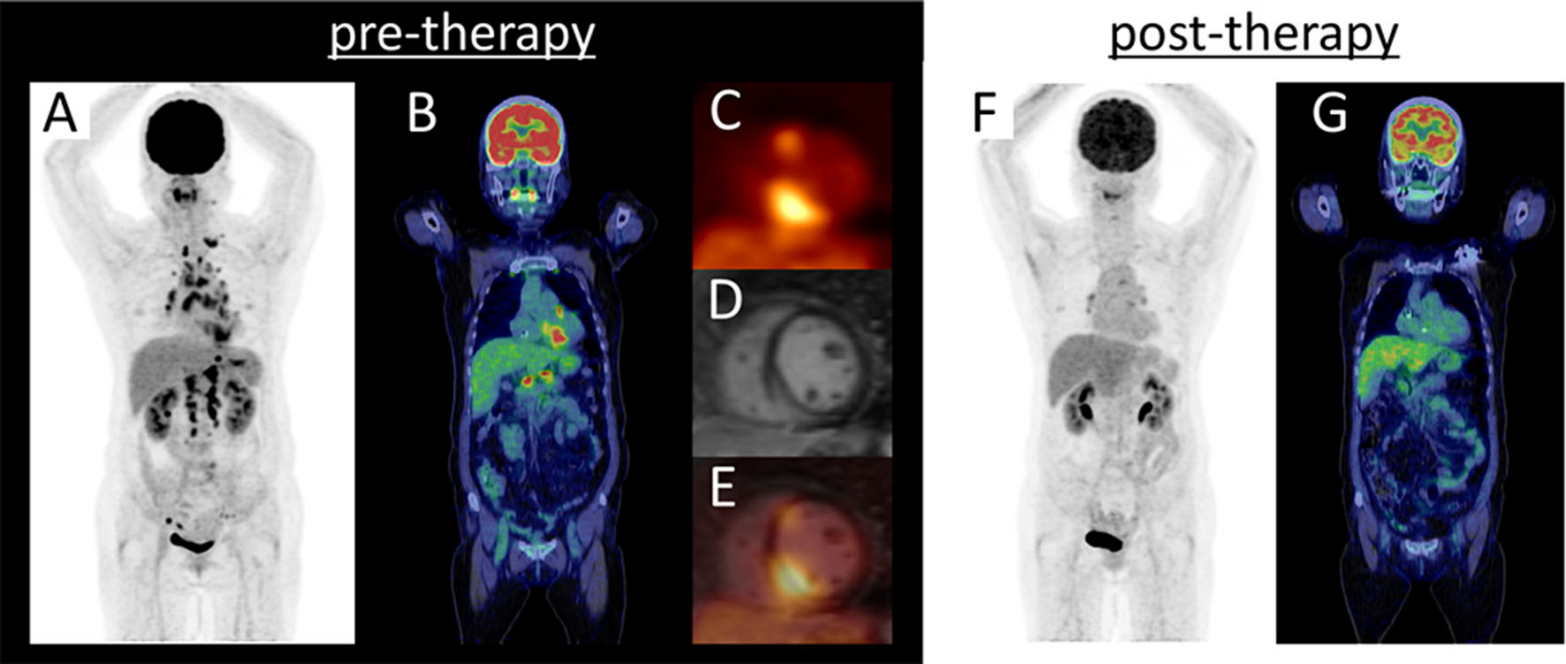
Representative case of cardiac sarcoidosis Maximum intensity projection (MIP) image of ^18^F-FDG PET (**A**), PET/CT coronal image (**B**), short-axis image of ^18^F-FDG PET (**C**), late gadolinium enhancement (LGE)-MRI (**D**), and fused image of ^18^F-FDG PET and LGE-MRI (**E**) at pre-therapy, MIP image of ^18^F-FDG PET (**F**) and PET/CT coronal image (**G**) at post-therapy (steroid 30 mg/1 month) are displayed. ^18^F-FDG PET detected focal cardiac uptake and multiple lymph node disease in the supraclavicular, mediastinum, hilum, abdominal, and pelvis region at pre-therapy. ^18^F-FDG uptake is seen at the same site of LGE-MRI abnormal intensity. At post-therapy, ^18^F-FDG uptakes were markedly lower. ^18^F-FDG is useful not only for diagnosis but also to confirm the effectiveness of treatments

Glucose is consumed in large quantities in the inflammatory process, and therefore active inflammatory lesions show high ^18^F-FDG accumulations. It is necessary to suppress physiological myocardial glucose metabolism in order to accurately evaluate myocardial inflammatory lesions using ^18^F-FDG PET. Among effective approaches to reducing physiological myocardial glucose metabolism, long-period fasting is the most common. Long-period fasting combined with a low-carbohydrate diet and/or high-fat diet and unfractionated heparin intravenous injection are also used. These approaches lead to myocardial free fatty acid metabolism dominance.^170^ ^18^F-FDG PET is more useful than are perfusion SPECT and delayed enhanced cardiac magnetic resonance (CMR) to not only diagnose but also monitor treatment effects in inflammatory heart disease such as cardiac sarcoidosis (Figure 10).^171^ Myocardial ischemia (reflecting a shift to glucose metabolism), other cardiomyopathy (reflecting microcirculatory ischemia and inflammation), and cardiac tumors also show ^18^F-FDG accumulation.^172^–^175^

Incomplete suppression of physiological myocardial ^18^F-FDG uptake may cause false positives. Therefore, new tracers have been developed to detect inflammatory heart disease and atherosclerotic lesions. These radiopharmaceuticals target tissue apoptosis, tissue calcification, activated macrophages, and tissue hypoxia.

^68^Ga complexed with [1,4,7,10-tetraazacyclododecane-1,4,7,10-tetraacetic acid]-1-Nal^3^-octreotide (^68^Ga-DOTANOC),^176^ fluorine-18 fluorothymidine (^18^F-FLT),^177^ ^68^Ga complexed with [1,4,7,10-tetraazacyclododecane-1,4,7,10-tetraacetic acid]-Phe^1^-Tyr^3^-octreotide (^68^Ga-DOTATOC),^178^ and fluorine-18 fluoromisonidazole (^18^F-FMISO)^179^ have been reported to improve specificity with regard to diagnosis of cardiac sarcoidosis.

^68^Ga-tagged tracers can be prepared using a generator system and have been applied for clinical oncology imaging. Activated macrophages show upregulated G-protein-coupled somatostatin receptor subtype-2 receptors. In an observational study involving oncology patients, uptake of ^68^Ga complexed with a somatostatin analog, 1,4,7,10-tetraazacyclododecane-1,4,7,10-tetraacetic acid-d-Phe^1^-Tyr^3^-octreotate (^68^Ga-DOTATATE), in large arteries increased in relation to age.^180^ A recent study prospectively revealed ^68^Ga-DOTATATE uptakes in carotid and coronary arteries in patients with unstable CVD.^181^ Unlike ^18^F-FDG, ^68^Ga-DOTATATE does not have physiological myocardial uptake and therefore could potentially play a clinical role in detecting vulnerable plaque.

An alternative to ^68^Ga, Copper-64 (^64^Cu) complexed with the somatostatin analog (^64^Cu-DOTATATE) has been used. ^64^Cu has a shorter positron range and longer half-life. Thus, ^64^Cu DOTATATE may have improved spatial resolution over that of ^68^Ga-DOTATATE. ^64^Cu DOTATATE also showed positive uptake in carotid atherosclerotic lesions.^182^ ^64^Cu-labeled DOTATATE uptake was positively linked to the expression of membrane receptor CD163, indicating that ^64^Cu-labeled DOTATATE uptake was associated with hemorrhagic macrophage migration.

#### Translocator protein

**Figure 11:**
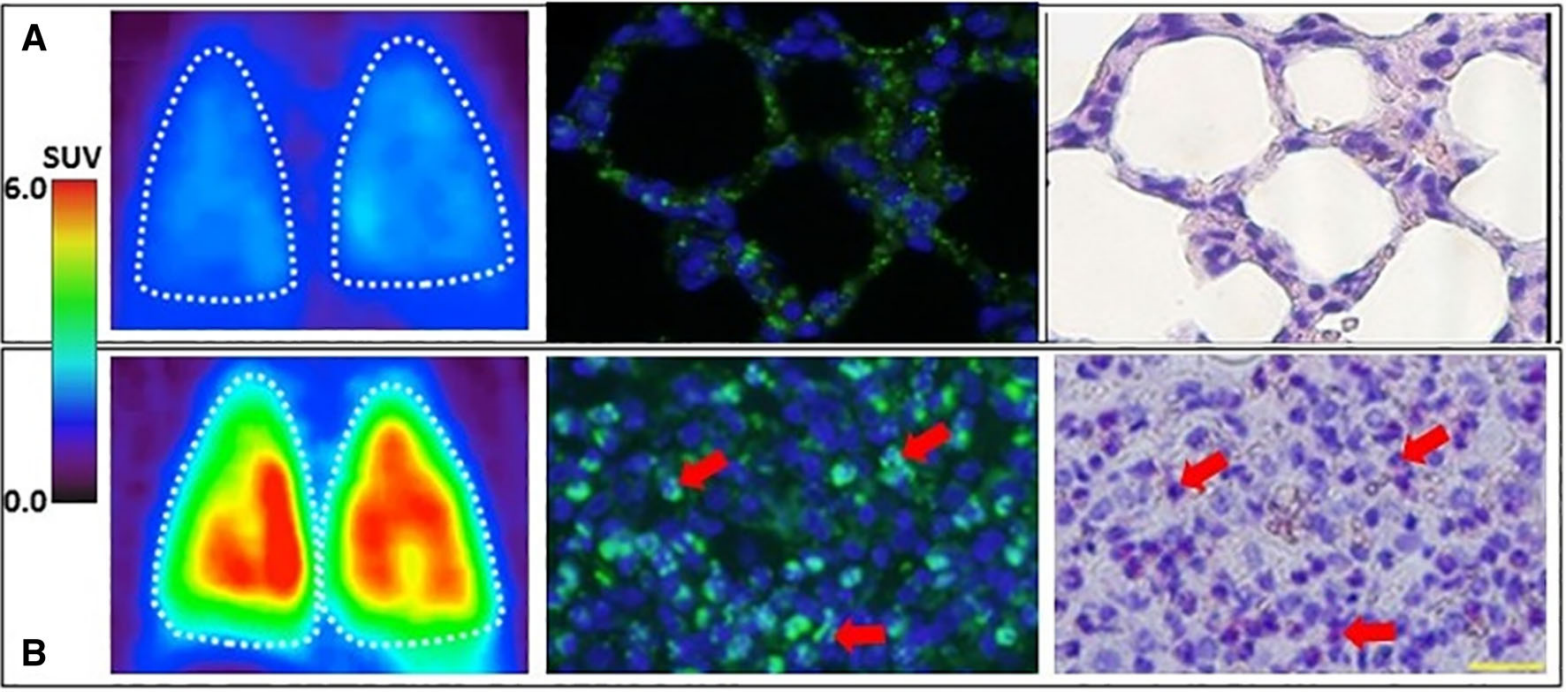
^18^F-FEDAC imaging a comparison between ^18^F-FEDAC imaging and double staining of translocator protein (TSPO) for neutrophils. Arrows indicate examples of cells doubly positive for TSPO (green) and chloroacetate esterase (red spots) staining. Control group showed no positive ^18^F-FEDAC uptake in either lung (**A**). No neutrophils were seen in the control. Lung injury model using lipopolysaccharide showed positive ^18^F-FEDAC uptake in both lungs (**B**)

**Figure 12:**
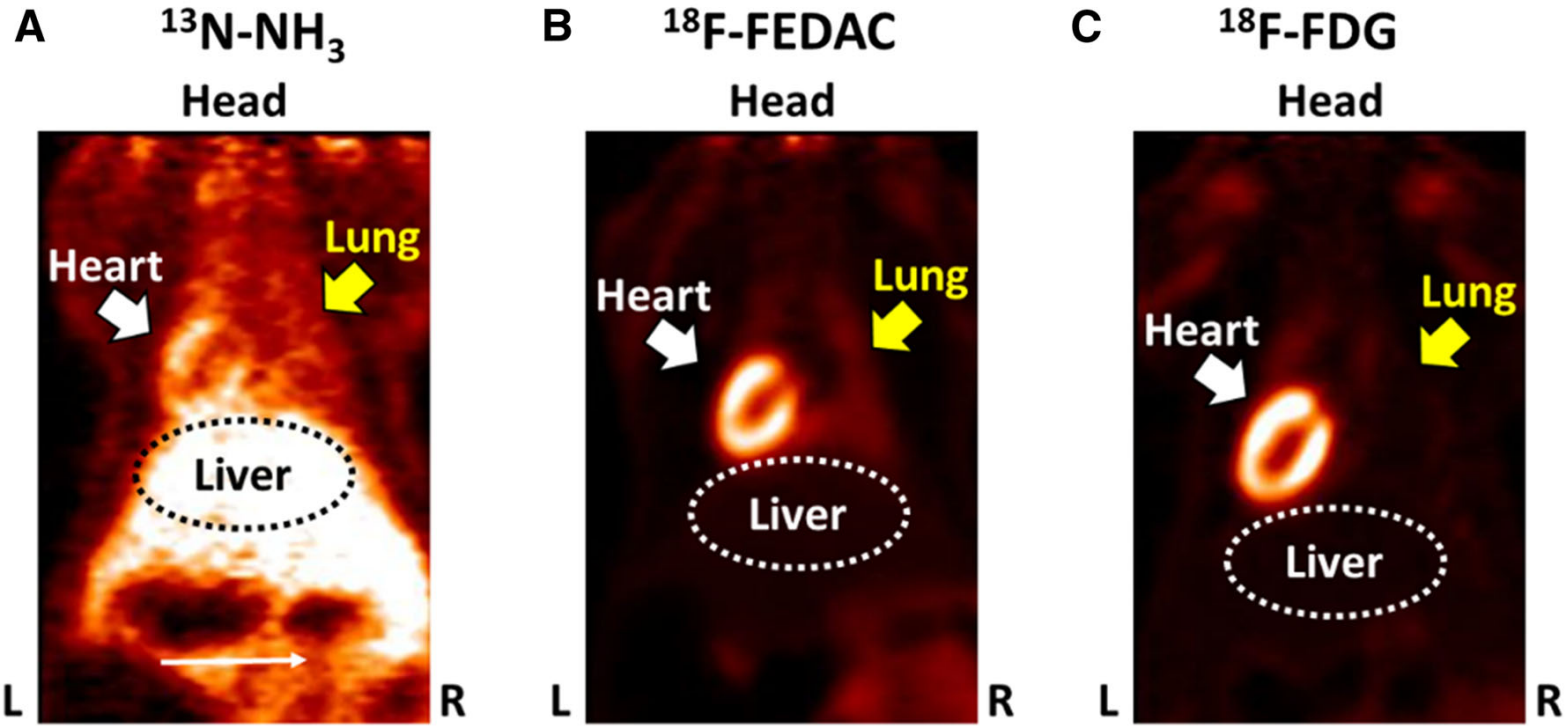
Histology showed leukocyte infiltration in the lung injury model. Scale bar: 20 µm. ^18^F-FEDAC showed higher uptake ratios of heart/lung and heart/liver compared to those with ^13^N-NH_3_ and similar to that with ^18^F-FDG. ^*18*^ *F-FDG*, ^18^F-fluorodeoxyglucose; ^*18*^ *F-FEDAC*, *N*-benzyl-*N*-methyl-2-[7,8-dihydro-7-(2-[^18^F]-fluoroethyl)-8-oxo-2-phenyl-9*H*-purin-9-yl] acetamide

Translocator protein 18kDa (TSPO), a peripheral-type benzodiazepine receptor, locates in peripheral tissue and the brain.^183^ TSPO is a protein highly expressed in activated cells of the mononuclear phagocyte lineage.^184^ Carbon-11 labeled [1-(2-chlorophenyl)-N-methyl-N-1(1-methylpropyl)-3-isoquinolinecarboxamide] (^11^C-PK11195) is a first specific ligand for TPSO, and its uptake has been revealed in symptomatic carotid atherosclerotic lesions.^185^ However, ^11^C-PK11195 has some limitations such as high non-specific binding and high lipophilicity. To overcome these limitations, we developed an ^18^F-labeled TPSO ligand, *N*-benzyl-*N*-methyl-2-[7,8-dihydro-7-(2-[^18^F]fluoroethyl)-8-oxo-2-phenyl-9*H*-purin-9-yl] acetamide (^18^F-FEDAC). ^18^F-FEDAC showed high in vitro binding affinity for TSPO with high selectivity.^186^ ^18^F-FEDAC was initially developed as a tracer for imaging brain inflammation, and subsequent study revealed that this tracer could potentially be used for imaging inflammation in peripheral organs.^187^ Indeed, ^18^F-FEDAC can be used to visualize lesions in rat liver.^14^,^188^ In a rat lung injury model, ^18^F-FEDAC uptake increased with the progression of lung inflammation (Figure 11).^189^ The uptake of ^18^F-FEDAC in the heart of a rat was approximately twice as high as that in the lung.^187^ With ^18^F-FEDAC the uptake ratio for heart to lung is higher than that with ^13^N-NH_3_. The same is true for the heart-to-liver uptake ratio measured with each of these tracers respectively. However, uptake ratios are similar for heart to lung and heart to liver measured using ^18^F-FEDAC and ^18^F-FDG (Figure 12). In this regard, ^18^F-FEDAC may have potential for detecting cardiac inflammatory lesions or vascular inflammatory lesions.

Fluorine-18 anion (^18^F^−^), which is administered as the sodium salt ^18^F-NaF, has been used as a bone-imaging agent to detect metastatic bone lesions. Since ^18^F^−^ accumulates in calcification lesions, it has also been used to evaluate the severity or disease activity of aortic stenosis.^190^ During the progression of atherosclerosis, calcification may appear in intermediate lesions. In contrast, with inflammation, active calcification may appear during the later stages of disease progression. However, it is still important to detect actively progressing calcification, because this may be one of the signs of plaque rupture.^191^ Prospective studies with clinical outcomes are ongoing to assess whether coronary ^18^F uptake represents a future cardiovascular risk.

## Summary and Conclusion

Nuclear cardiology using targeted tracers via SPECT and PET allows for diagnosis through non-invasive imaging. Not only myocardial perfusion but also cardiac metabolism, sympathetic nervous system activity, and inflammatory disease are targeted by nuclear cardiology using specific radiopharmaceuticals.

## Electronic supplementary material

Below is the link to the electronic supplementary material.

Supplementary material 1 (PPTX 1515 kb)

## Abbreviations

^11^C-CGP12177: (*S*)-4-(3-((1,1-dimethylethyl)amino)-2-hydroxypropoxy)-[*carbonyl*-^11^C]1,3-dihydro-2*H*-benzimidazol-2-one
^11^C-CGP12388: (*S*)-4-(3-(2’-[*methine*-^11^C]isopropylamino)-2-hydroxypropoxy)-2*H*-benzimidazol-2-one
^11^C-HED: [^11^C]hydroxyephedrine
^11^C-PK11195: 1-(2-chlorophenyl)-*N*-[^11^C]methyl-*N*-1(1-methylpropyl)-3-isoquinolinecarboxamide
^123^I-BMIPP: β-methyl-*p*-[^123^I]iodophenylpentadecanoic acid
^123^I-IPPA: *p*-/*o*-[^123^I]iodophenylpentadecanoic acid
^123^I-MIBG: *m*-[^123^I]iodobenzylguanidine
^13^N-NH_3_: [^13^N]ammonia
^15^O-H_2_O: [^15^O]water
^18^F-FDG: 2-[^18^F]fluorodeoxyglucose
^18^F-FEDAC: *N*-benzyl-*N*-methyl-2-[7,8-dihydro-7-(2-[^18^F]fluoroethyl)-8-oxo-2-phenyl-9*H*-purin-9-yl]acetamide
^18^F-FLT: [^18^F]fluorothymidine
^18^F-FMISO: [^18^F]fluoromisonidazole
^18^F-FTHA: [^18^F]fluoro-6-thia-heptadecanoic acid
^18^F-NaF: Sodium [^18^F]fluoride
^68^Ga-DOTANOC: ^68^Ga-complex with 1,4,7,10-tetraazacyclododecane-1,4,7,10-tetraacetic acid-1-Nal^3^-octreotide
^68^Ga-DOTATATE: ^68^Ga-complex with 1,4,7,10-tetraazacyclododecane-1,4,7,10-tetraacetic acid-d-Phe^1^-Tyr^3^-octreotate
^68^Ga-DOTATOC: ^68^Ga-complex with 1,4,7,10-tetraazacyclododecane-1,4,7,10-tetraacetic acid-d-Phe^1^-Tyr^3^-octreotide
^99m^Tc-MIBI: ^99m^Tc-sestamibi
acetyl-CoA: Acetyl coenzyme A
Ach: Acetylcholine
ATP: Adenosine triphosphate
CVD: Cardiovascular disease
CMR: Cardiac magnetic resonance
COMT: Catechol-*O*-methyltransferase
ECG: Electrocardiographically
FDA: Food and Drug Administration
FFA: Free fatty acid
HFpEF: Heart failure with preserved ejection fraction
HF: Heart failure
HMR: Heart-to-mediastinum ratio
HR: Heart rate
ICD: Indication for cardioverter-defibrillator implantation
LMI1195: *N*-(3-bromo-4-(3-[^18^F]fluoropropoxy)benzyl)-guanidine
LV: Left ventricular
MAO: Monoamine oxidase
MBF: Myocardial blood flow
MPI: Myocardial perfusion imaging
NE: Norepinephrine
PAP: Pulmonary artery pressure
PET: Positron emission tomography
PH: Pulmonary hypertension
PS: Phosphatidylserine
RV: Right ventricle
SPECT: Single-photon emission computed tomography
TCA: Tricarboxylic acid
TSPO: Translocator protein 18kDa
VMAT: Vesicular monoamine transporter
WBC: White blood cell
β-AR: β-adrenergic receptor
