# Supplementary material for: Radiopharmaceutical tracers for cardiac imaging
Source: J Nucl Cardiol. 2017 Dec 1;25(4):1204–36. doi: 10.1007/s12350-017-1131-5 (PMC6133155; doi:10.1007/s12350-017-1131-5)
Supplement: Supplementary file 1 — Supplementary material 1 (PPTX 1515 kb) [file 12350_2017_1131_MOESM1_ESM.pptx]

## Slide 1
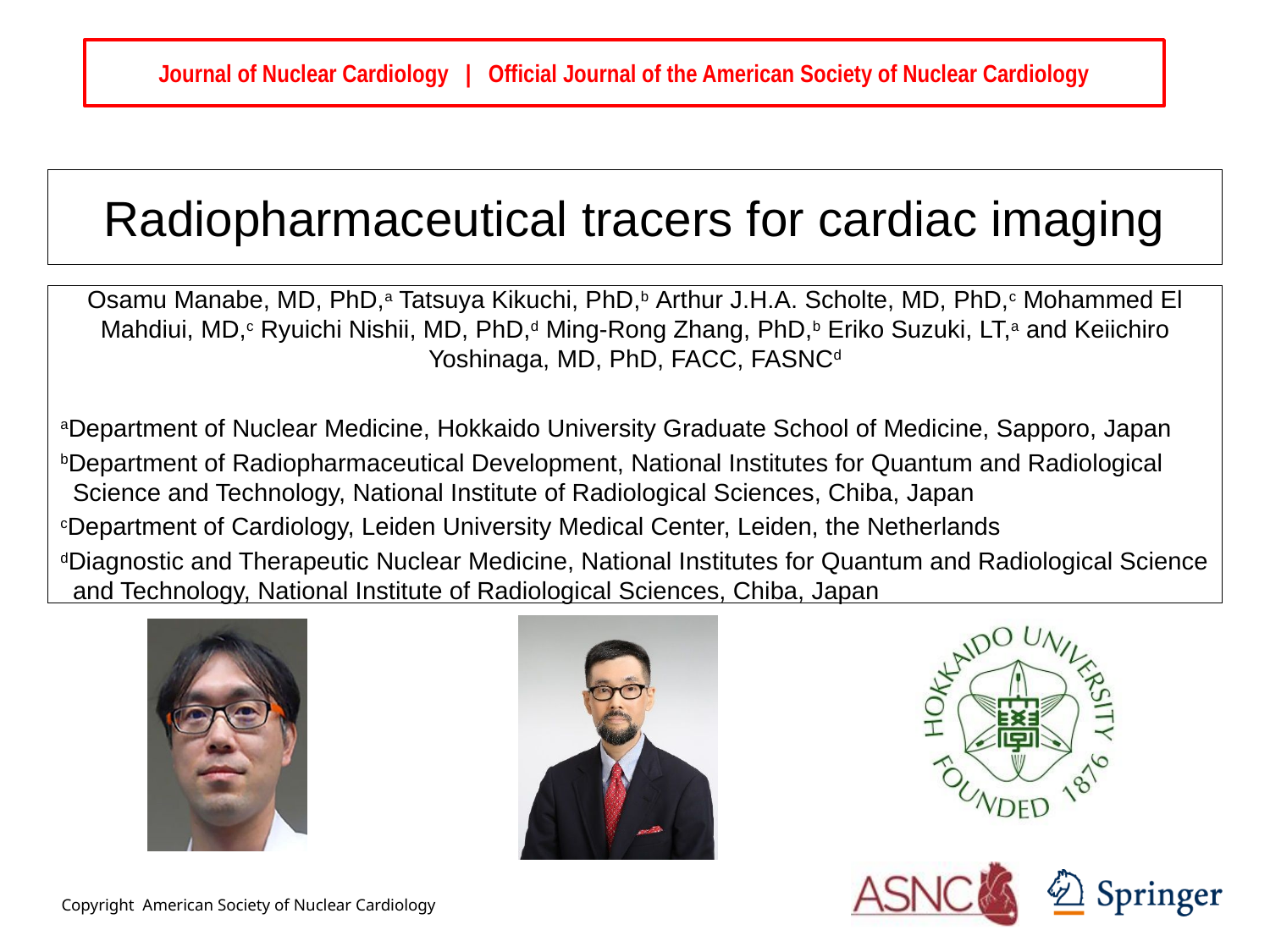

Journal of Nuclear Cardiology | Official Journal of the American Society of Nuclear Cardiology
# Radiopharmaceutical tracers for cardiac imaging
Osamu Manabe, MD, PhD,a Tatsuya Kikuchi, PhD,b Arthur J.H.A. Scholte, MD, PhD,c Mohammed El Mahdiui, MD,c Ryuichi Nishii, MD, PhD,d Ming-Rong Zhang, PhD,b Eriko Suzuki, LT,a and Keiichiro Yoshinaga, MD, PhD, FACC, FASNCd
aDepartment of Nuclear Medicine, Hokkaido University Graduate School of Medicine, Sapporo, Japan
bDepartment of Radiopharmaceutical Development, National Institutes for Quantum and Radiological Science and Technology, National Institute of Radiological Sciences, Chiba, Japan
cDepartment of Cardiology, Leiden University Medical Center, Leiden, the Netherlands
dDiagnostic and Therapeutic Nuclear Medicine, National Institutes for Quantum and Radiological Science and Technology, National Institute of Radiological Sciences, Chiba, Japan
Copyright American Society of Nuclear Cardiology

## Slide 2
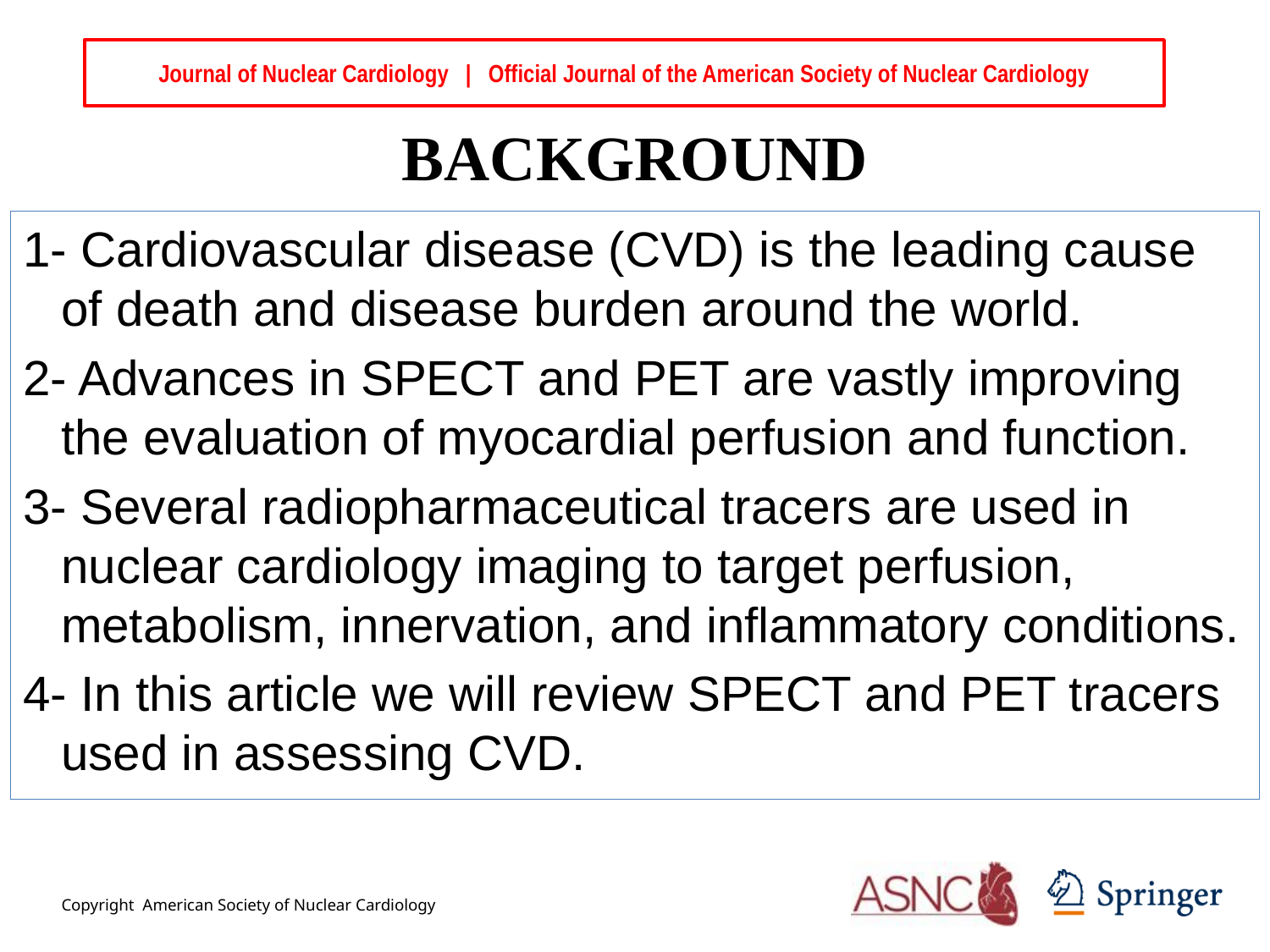

Journal of Nuclear Cardiology | Official Journal of the American Society of Nuclear Cardiology
# BACKGROUND
1- Cardiovascular disease (CVD) is the leading cause of death and disease burden around the world.
2- Advances in SPECT and PET are vastly improving the evaluation of myocardial perfusion and function.
3- Several radiopharmaceutical tracers are used in nuclear cardiology imaging to target perfusion, metabolism, innervation, and inflammatory conditions.
4- In this article we will review SPECT and PET tracers used in assessing CVD.
Copyright American Society of Nuclear Cardiology

## Slide 3
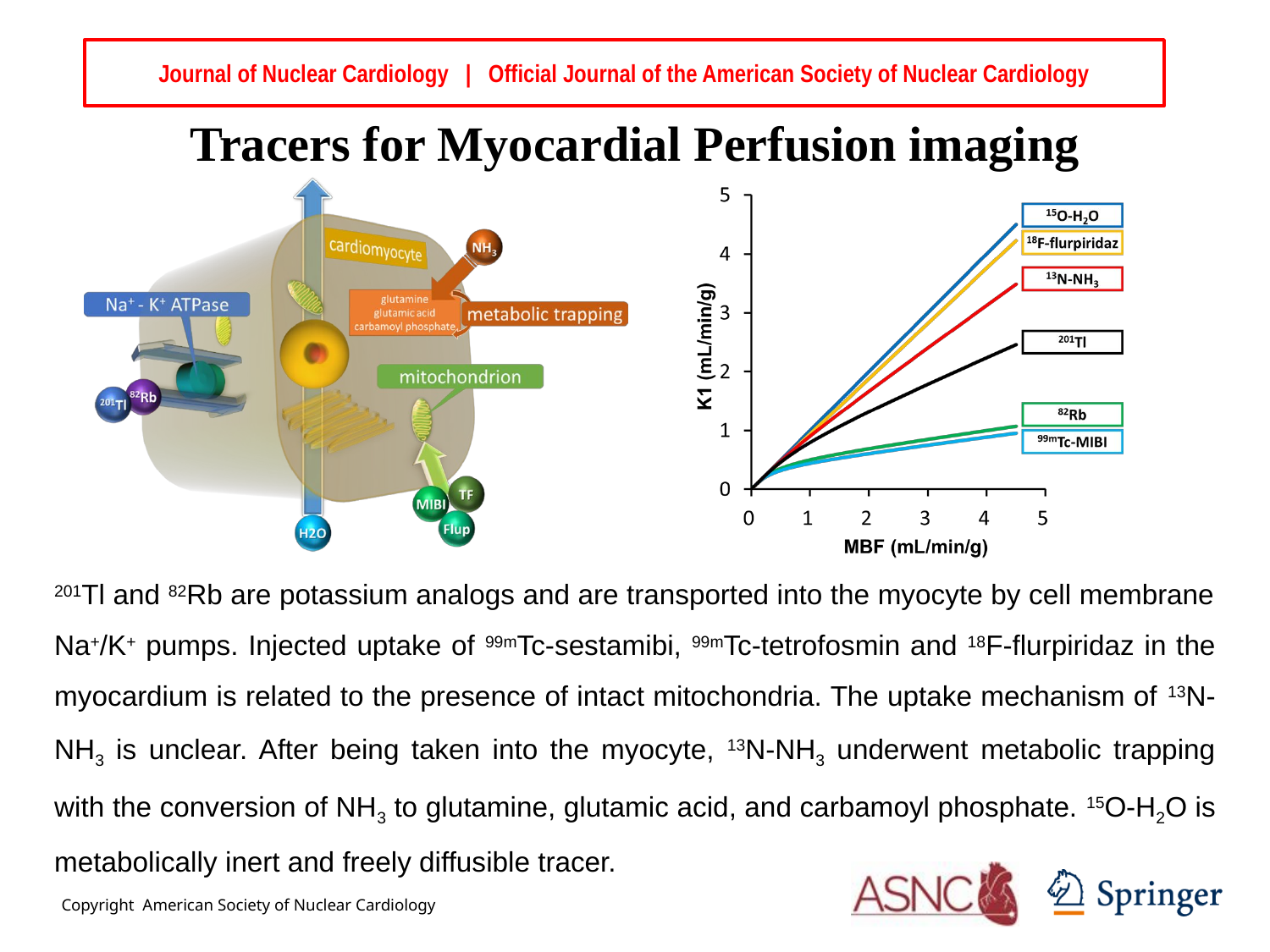

Journal of Nuclear Cardiology | Official Journal of the American Society of Nuclear Cardiology
# Tracers for Myocardial Perfusion imaging
201Tl and 82Rb are potassium analogs and are transported into the myocyte by cell membrane Na+/K+ pumps. Injected uptake of 99mTc-sestamibi, 99mTc-tetrofosmin and 18F-flurpiridaz in the myocardium is related to the presence of intact mitochondria. The uptake mechanism of 13N-NH3 is unclear. After being taken into the myocyte, 13N-NH3 underwent metabolic trapping with the conversion of NH3 to glutamine, glutamic acid, and carbamoyl phosphate. 15O-H2O is metabolically inert and freely diffusible tracer.
Copyright American Society of Nuclear Cardiology

## Slide 4
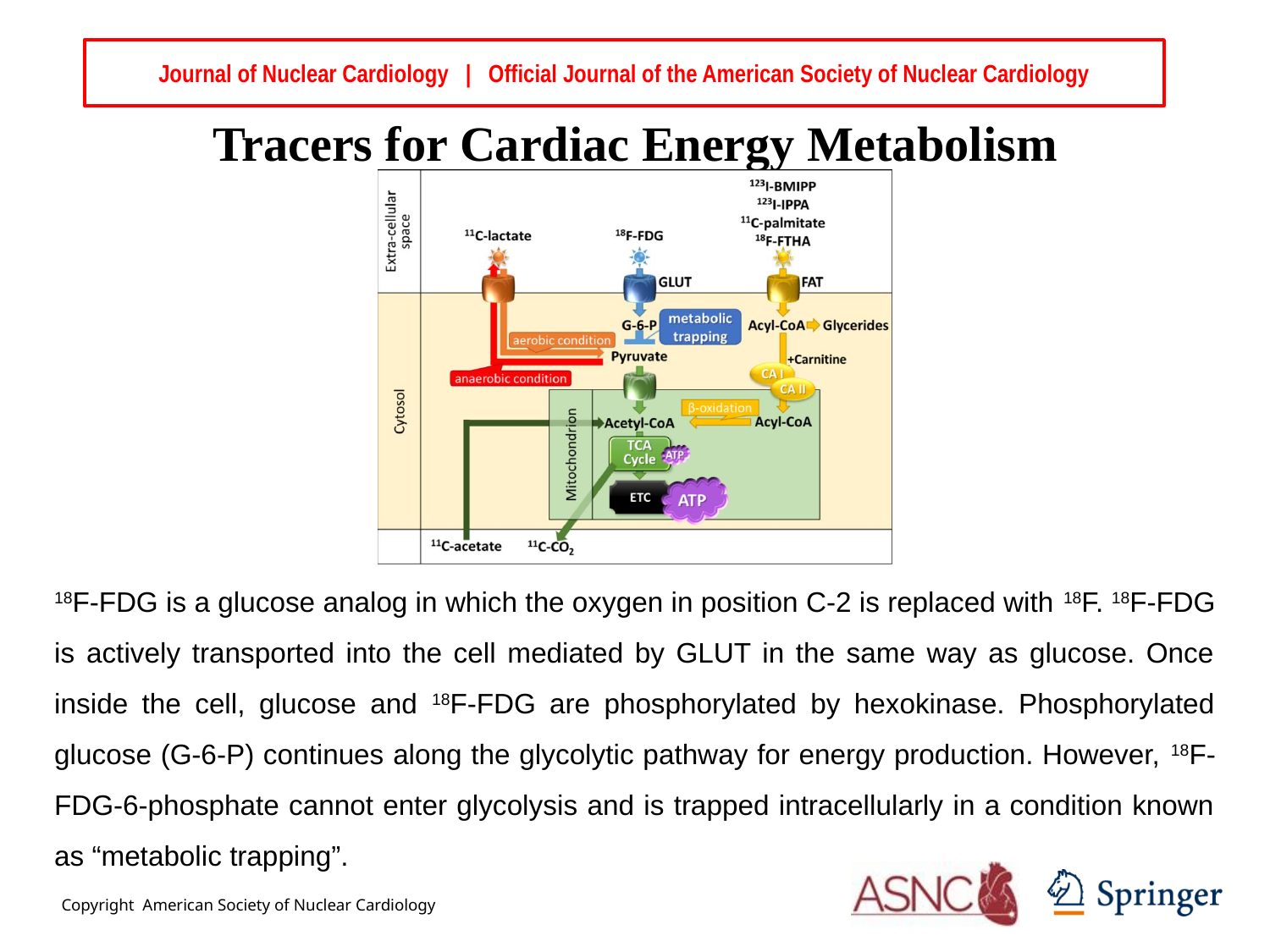

Journal of Nuclear Cardiology | Official Journal of the American Society of Nuclear Cardiology
# Tracers for Cardiac Energy Metabolism
18F-FDG is a glucose analog in which the oxygen in position C-2 is replaced with 18F. 18F-FDG is actively transported into the cell mediated by GLUT in the same way as glucose. Once inside the cell, glucose and 18F-FDG are phosphorylated by hexokinase. Phosphorylated glucose (G-6-P) continues along the glycolytic pathway for energy production. However, 18F-FDG-6-phosphate cannot enter glycolysis and is trapped intracellularly in a condition known as “metabolic trapping”.
Copyright American Society of Nuclear Cardiology

## Slide 5
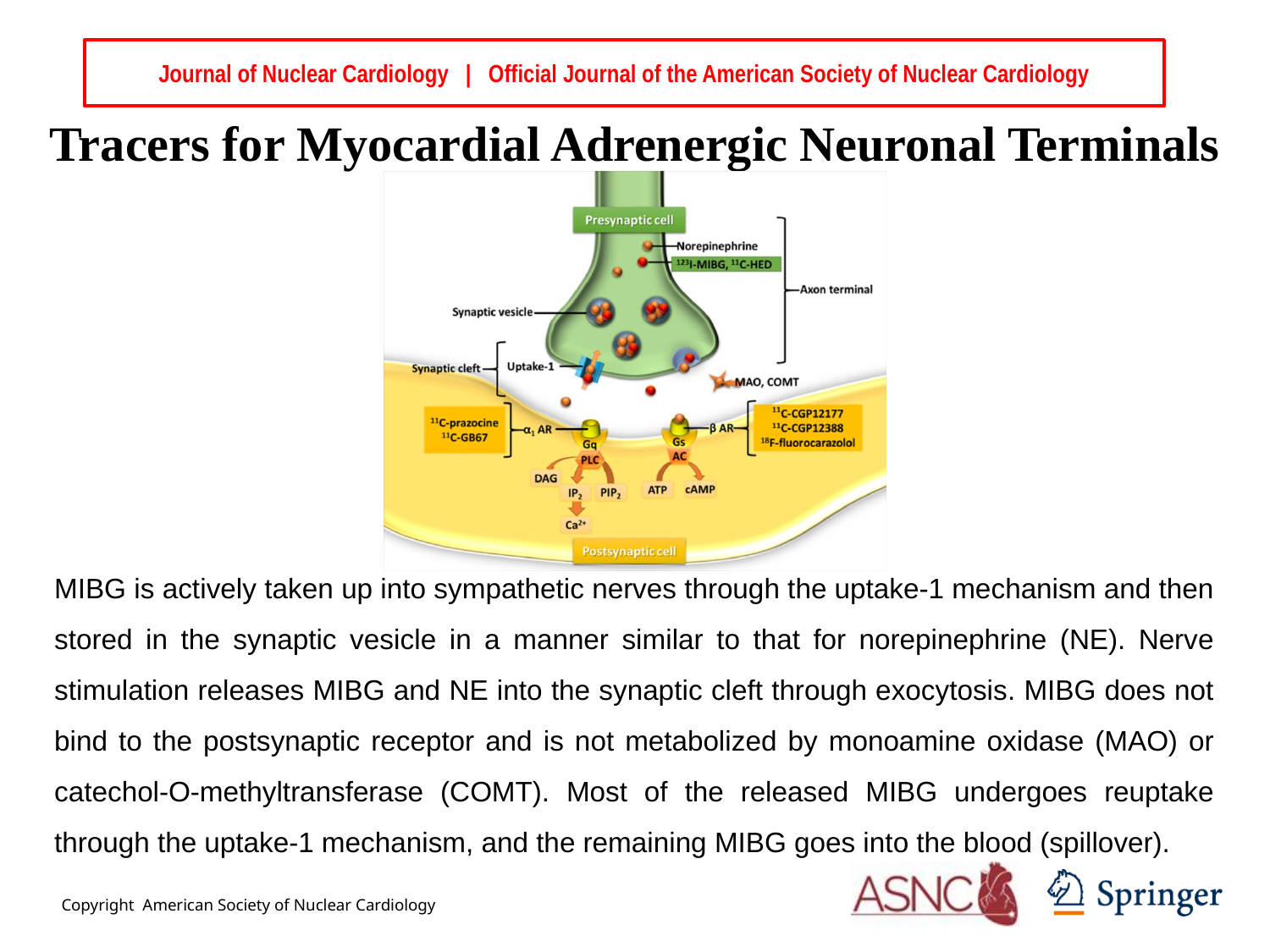

Journal of Nuclear Cardiology | Official Journal of the American Society of Nuclear Cardiology
# Tracers for Myocardial Adrenergic Neuronal Terminals
MIBG is actively taken up into sympathetic nerves through the uptake-1 mechanism and then stored in the synaptic vesicle in a manner similar to that for norepinephrine (NE). Nerve stimulation releases MIBG and NE into the synaptic cleft through exocytosis. MIBG does not bind to the postsynaptic receptor and is not metabolized by monoamine oxidase (MAO) or catechol-O-methyltransferase (COMT). Most of the released MIBG undergoes reuptake through the uptake-1 mechanism, and the remaining MIBG goes into the blood (spillover).
Copyright American Society of Nuclear Cardiology

## Slide 6
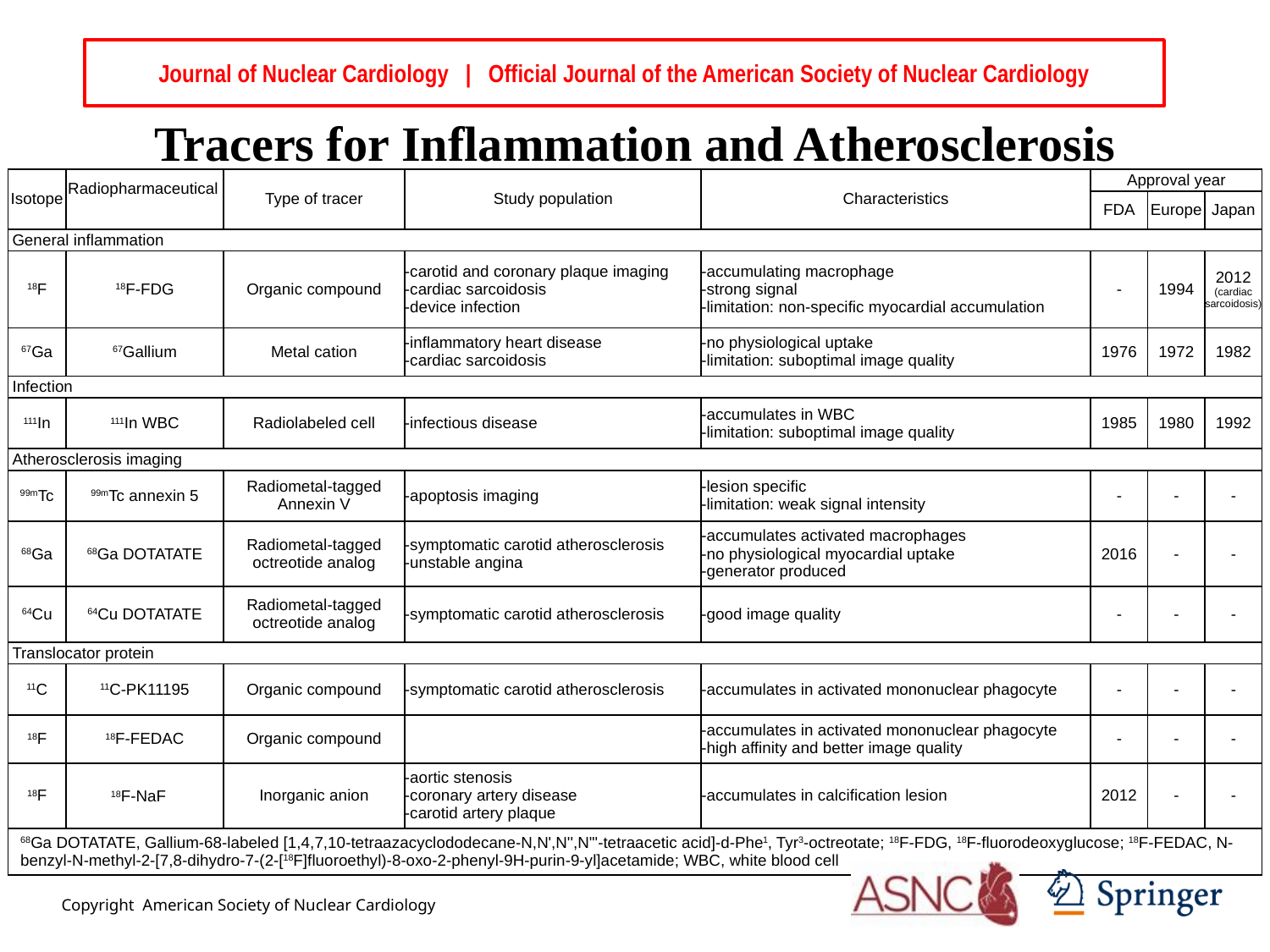

Journal of Nuclear Cardiology | Official Journal of the American Society of Nuclear Cardiology
# Tracers for Inflammation and Atherosclerosis
| Isotope | Radiopharmaceutical | Type of tracer | Study population | Characteristics | Approval year | | |
| --- | --- | --- | --- | --- | --- | --- | --- |
| | | | | | FDA | Europe | Japan |
| General inflammation | | | | | | | |
| 18F | 18F-FDG | Organic compound | -carotid and coronary plaque imaging -cardiac sarcoidosis -device infection | -accumulating macrophage -strong signal -limitation: non-specific myocardial accumulation | - | 1994 | 2012 (cardiac sarcoidosis) |
| 67Ga | 67Gallium | Metal cation | -inflammatory heart disease -cardiac sarcoidosis | -no physiological uptake -limitation: suboptimal image quality | 1976 | 1972 | 1982 |
| Infection | | | | | | | |
| 111In | 111In WBC | Radiolabeled cell | -infectious disease | -accumulates in WBC -limitation: suboptimal image quality | 1985 | 1980 | 1992 |
| Atherosclerosis imaging | | | | | | | |
| 99mTc | 99mTc annexin 5 | Radiometal-tagged Annexin V | -apoptosis imaging | -lesion specific -limitation: weak signal intensity | - | - | - |
| 68Ga | 68Ga DOTATATE | Radiometal-tagged octreotide analog | -symptomatic carotid atherosclerosis -unstable angina | -accumulates activated macrophages -no physiological myocardial uptake -generator produced | 2016 | - | - |
| 64Cu | 64Cu DOTATATE | Radiometal-tagged octreotide analog | -symptomatic carotid atherosclerosis | -good image quality | - | - | - |
| Translocator protein | | | | | | | |
| 11C | 11C-PK11195 | Organic compound | -symptomatic carotid atherosclerosis | -accumulates in activated mononuclear phagocyte | - | - | - |
| 18F | 18F-FEDAC | Organic compound | | -accumulates in activated mononuclear phagocyte -high affinity and better image quality | - | - | - |
| 18F | 18F-NaF | Inorganic anion | -aortic stenosis -coronary artery disease -carotid artery plaque | -accumulates in calcification lesion | 2012 | - | - |
| 68Ga DOTATATE, Gallium-68-labeled [1,4,7,10-tetraazacyclododecane-N,N',N'',N'''-tetraacetic acid]-d-Phe1, Tyr3-octreotate; 18F-FDG, 18F-fluorodeoxyglucose; 18F-FEDAC, N-benzyl-N-methyl-2-[7,8-dihydro-7-(2-[18F]fluoroethyl)-8-oxo-2-phenyl-9H-purin-9-yl]acetamide; WBC, white blood cell | | | | | | | |
Copyright American Society of Nuclear Cardiology

## Slide 7
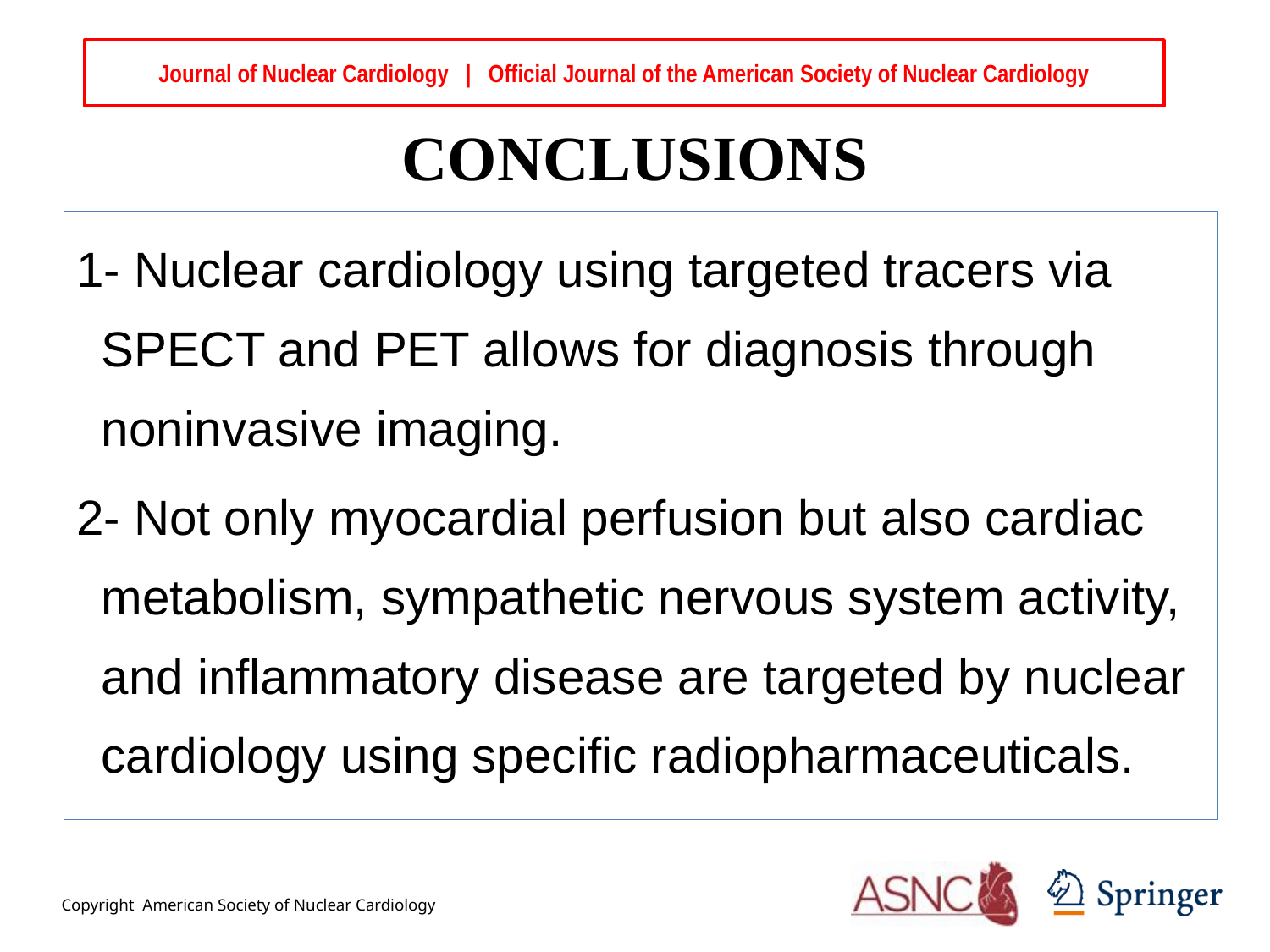

Journal of Nuclear Cardiology | Official Journal of the American Society of Nuclear Cardiology
# CONCLUSIONS
1- Nuclear cardiology using targeted tracers via SPECT and PET allows for diagnosis through noninvasive imaging.
2- Not only myocardial perfusion but also cardiac metabolism, sympathetic nervous system activity, and inflammatory disease are targeted by nuclear cardiology using specific radiopharmaceuticals.
Copyright American Society of Nuclear Cardiology
